# Synchronization of Two Homodromy Rotors Installed on a Double Vibro-Body in a Coupling Vibration System

**DOI:** 10.1371/journal.pone.0126069

**Published:** 2015-05-19

**Authors:** Pan Fang, Yongjun Hou, Yanghai Nan

**Affiliations:** 1 School of Mechanical Engineering, Southwest Petroleum University, Chengdu, China; 2 Department of Mechanical Engineering and Robotics, Universite Libre de Bruxelles, Brussel, Belgium; Hong Kong Baptist University, CHINA

## Abstract

A new mechanism is proposed to implement synchronization of the two unbalanced rotors in a vibration system, which consists of a double vibro-body, two induction motors and spring foundations. The coupling relationship between the vibro-bodies is ascertained with the Laplace transformation method for the dynamics equation of the system obtained with the Lagrange’s equation. An analytical approach, the average method of modified small parameters, is employed to study the synchronization characteristics between the two unbalanced rotors, which is converted into that of existence and the stability of zero solutions for the non-dimensional differential equations of the angular velocity disturbance parameters. By assuming the disturbance parameters that infinitely approach to zero, the synchronization condition for the two rotors is obtained. It indicated that the absolute value of the residual torque between the two motors should be equal to or less than the maximum of their coupling torques. Meanwhile, the stability criterion of synchronization is derived with the Routh-Hurwitz method, and the region of the stable phase difference is confirmed. At last, computer simulations are preformed to verify the correctness of the approximate solution of the theoretical computation for the stable phase difference between the two unbalanced rotors, and the results of theoretical computation is in accordance with that of computer simulations. To sum up, only the parameters of the vibration system satisfy the synchronization condition and the stability criterion of the synchronization, the two unbalanced rotors can implement the synchronization operation.

## Introduction

The word “synchronization” is often encountered in both scientific and everyday language. Our surroundings are full of synchronization phenomenon, which is considered as an adjustment of rhythms of oscillating objects due to their internal weak couplings. For examples: violinists play in unison; insects in a population emit acoustic or light pulses with a common rate; birds in a flock flap their wings simultaneously; the heart of a rapidly galloping horse contracts once per locomotory cycle. Huygens firstly described the notion of the synchronization by experiments that two pendulum clocks hung on a common support in 1665 [1]. Pol showed that the frequency of a generator can be entrained, or synchronized, by a weak external signal of a slightly different frequency in 1920 [2]. In the middle of the nineteenth century, Rayleigh [3] described the interesting phenomenon of synchronization in acoustical systems. The first English monograph related to the synchronization problems is written by Blekhman [4]; he primarily addressed mechanical oscillators, pendulum clocks in particular, systems with rotating elements, technological equipment, but also some electronic and quantum generator; many years later, he also investigated controlled synchronization of two vibroactuators based on a speed-gradient[5, 6]. Pikovsky issued his monograph that consider synchronization as a universal concept in nonlinear sciences and review classical results on the synchronization of periodic oscillators [7]. Zhang investigated the synchronization problem for a class of discrete-time complex-valued neural networks with time-varying delays [8]. Nowadays, the researchers mainly focus synchronization on physical, biological, chemical and social systems, etc. In physical systems, the most representatives are synchronization of complex networks and mechanical systems. For the synchronization of complex networks, Arenas reported the advances in the comprehension of synchronization phenomena when oscillating elements are constrained to interact in a complex network topology [9]. How the feedback from dynamical clusters can shape the network connection weights and an adaptive network spontaneously forms scale-free structure were explored by Yuan Wang[10, 11]. For the synchronization of pendulum clocks, Senator developed synchronization of two coupled, similarly sized, escapement-driven pendulum clocks [12]. Jovanovic studied two models of connected pendulum clocks synchronizing their oscillations, a phenomenon originally observed by Huygens, with the Poincare´ method, and they found that the in-phase linear mode damps out faster than the anti-phase mode [13]. Koluda considered two and multiple self-excited double pendula hanging from a horizontal beam with the energy balanced method, on which they found how the energy is transferred between the pendula via the oscillating beam allowing the pendula’ synchronization[14–16]. For synchronization of multiple coupling rotors, Wen employed the average method to study synchronization and stability of multiple unbalanced rotors hung on a vibro-body in vibration systems, and applied such synchronization theory to invent many synchronization machines [17]. Sperling presented analytical and numerical investigations of a two-plane automatic balancing device for equilibration of rigid-rotor unbalance [18]. Balthazar examined self-synchronization of four non-ideal exciters in non-linear vibration system via numerical simulations [19, 20]. Djanan explored the condition for which three motors working on a same plate can enter into synchronization with the phase difference depending on the physical characteristics of the motors and the plate, and it is indicated that one can obtain a reduction of vibration when the motors are different and rotates in opposite directions [21]. Zhao proposed the average method of modified small parameters to investigate the synchronization of more than two exciters in far vibration systems, which immensely simplify the process for solving the theory approximation solution [22–28]. Later, Fang applied Zhao’s method to investigate the self-synchronization of two homodromy rotors coupled with a pendulum rod in a far-resonant vibration system [29].

The above-mentioned research of the mechanical systems is mainly synchronization of the pendula or the rotors directly installed on a movable beam or vibro-body. In this paper, we consider the synchronization and the synchronization stability of two homodromy unbalanced rotors installed on two vibro-bodies, respectively. The synchronization implementation of the two rotors relies on the coupling springs between vibro-body 1 and 2. The performed approximate analytical analysis, building on the original work of Zhao Chunyu, allows deriving the synchronization condition and stability criterion and explaining the synchronization discipline with considering diversity features of the vibration system. Finally, some numerical simulations are performed to verify the correctness of the theoretical analysis.

This paper is organized as follows. The second section describes the considered model and dynamics equations of the vibration system. The third section we explain our method to derive the synchronization condition and the synchronization stability criterion of the system. The fourth section presents the results of our numerical simulations for the theoretical approximate solutions. The fifth section gives some computer simulations to verify our theoretical solutions. Finally, we summarize our results in the last section.

## Model description

**Fig 1:**
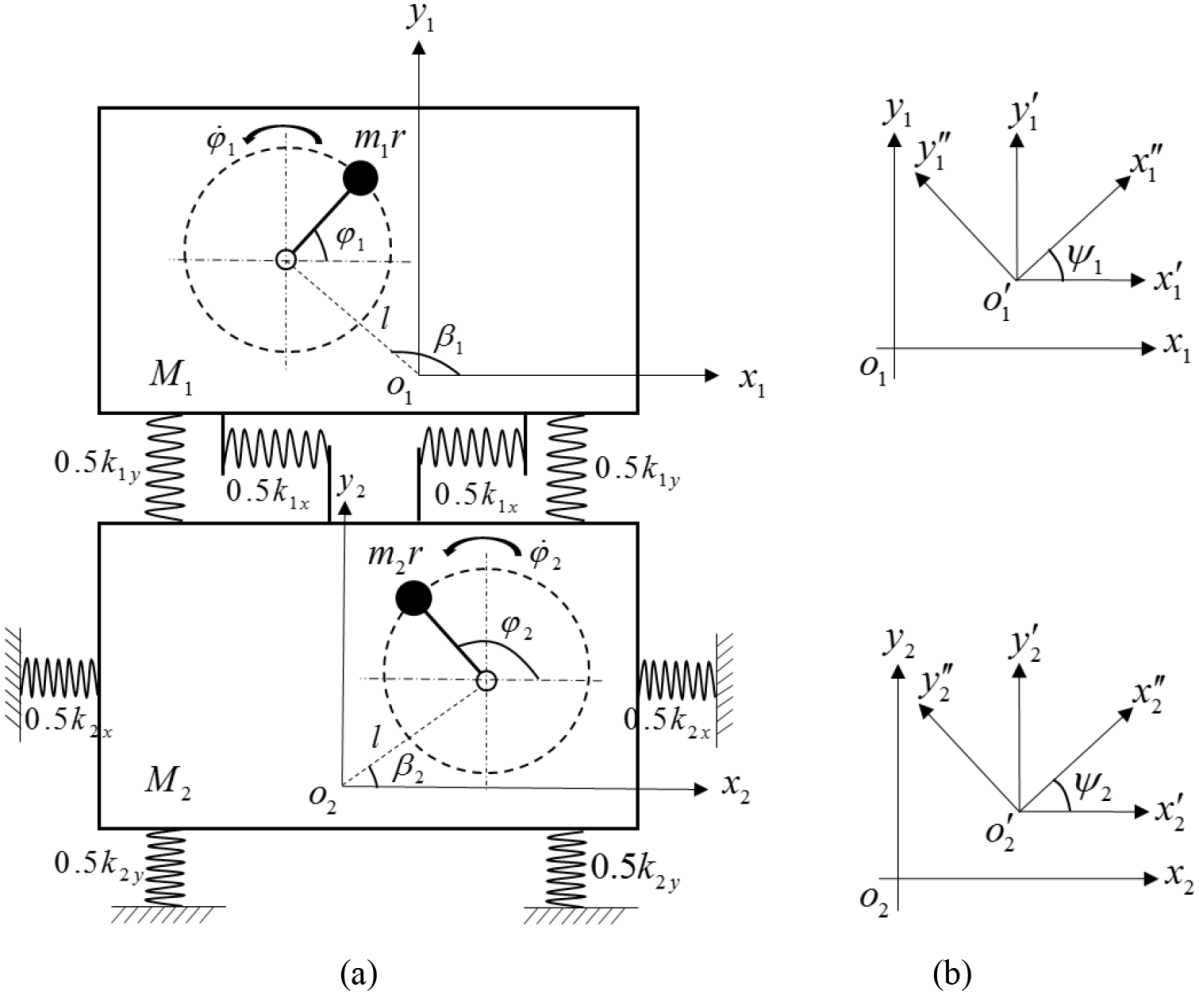
The model of the vibration system. (a) dynamic model of the double vibro-body system with two induction motors rotating in the same direction, (b) the reference frame system.

Fig 1 shows the dynamics model of the considered vibration system, which consists of two rigid vibro-bodies (vibro-body 1 and vibro-body 2), on which two induction motor are installed, respectively. Each of the vibro-body is supported on an elastic foundation consisting of four springs symmetrically installed. Rigid vibro-body 1 is connected with vibro-body 2 by some stronger stiffness springs (*k*_*x1*_, *k*_*y1*_, *k*_*ψ1*_), and vibro-body 1 is connected a fix foundation with some weaker stiffness springs (*k*_*x2*_, *k*_*y2*_, *k*_*ψ2*_). The two homodromy unbalanced rotors, driven separately by two induction motors, are installed in the vibro-bodies with the equal distance *l* from the rotation point of the rotor to the mass center of the vibro-body. During the starting process, three motors are supplied with the electric source at same time. The mass centers of the rigid vibro-body 1 and 2 are *o*_1_ and *o*_2_, respectively. As illustrated in Fig 1(b), six reference frames of the system can be assigned as follows: the fixed frames *o*_1_x_1_y_1_ and *o*_2_x_2_y_2_; the non-rotating moving frames ${o^{\prime }}_{1}{x^{\prime }}_{1}{y^{\prime }}_{1}$ and ${o^{\prime }}_{2}{x^{\prime }}_{2}{y^{\prime }}_{2}$, that undergoes the translation motion while remaining parallel to *o*_1_x_1_y_1_ and *o*_2_x_2_y_2_, respectively; the rotating frames ${o^{\prime }}_{1}{x^{\prime \prime }}_{1}{y^{\prime \prime }}_{1}$ and ${o^{\prime }}_{2}{x^{\prime \prime }}_{2}{y^{\prime \prime }}_{2}$, that dedicates the rotation motion around points ${o^{\prime }}_{1}$ and ${o^{\prime }}_{2}$, respectively. The six reference frames of the vibro-bodies separately coincide with each other when the system is in the static equilibrium state.

Since the two vibro-bodies are supported by two elastic foundations, it exhibits six degrees of freedom. The mass center coordinates of vibro-bodies x_1_, x_2_, y_1_ and y_2_, as well as the rotation coordinates *ψ*_*1*_ and *ψ*_*2*_, are set as the independent coordinates. The unbalanced rotors rotate about their own spin axes, which are denoted by *φ*_*1*_ and *φ*_*2*_, respectively.

In reference frame *o˝x˝y˝*, the coordinates of each exciter,${\Phi^{\prime \prime }}_{i}$ , can be described by

$${\Phi^{\prime \prime }}_{q}=\left[\begin{array}{c} l\cos\beta_{q}+r\cos\varphi_{q}\\ l\sin\beta_{q}+r\sin\varphi_{q} \end{array}\right],\;q=1,2$$ (1)

In reference frame *o´x´y´*, the coordinates of each exciter,${\Phi^{\prime }}_{i}$ , can be expressed by

$${\Phi^{\prime }}_{q}=\Gamma {\Phi^{\prime \prime }}_{q},\;\Gamma =\left[\begin{array}{cc} \cos\psi_{q} & \sin\psi_{q}\\ -\sin\psi_{q} & \cos\psi_{q} \end{array}\right]\;,\;q=1,2$$ (2)

In reference frame *oxy*, the coordinates of each exciter, **Φ**_*i*_, can be written by

$$\Phi_{q}=\Phi_{0}+\Gamma {\Phi^{\prime }}_{q}\;,\;\Phi_{0}={\left[x_{q},y_{q}\right]}^{\text{T}},\;q=1,2$$ (3)

The kinetic energy of the vibration system can be expressed as

$$T=\frac{1}{2}\sum_{q=1}^{2}M_{q}(x_{q}^{2}+y_{q}^{2})+\frac{1}{2}\sum_{q=1}^{2}J_{q}\psi_{q}^{2}+\frac{1}{2}\sum_{q=1}^{2}J_{0q}\varphi_{q}^{2}+\frac{1}{2}\sum_{q=1}^{2}m_{q}{\dot{\Phi }}^{\text{T}}{}_{q}\Phi_{q}$$ (4)

During the operation process of the vibration system, the position vector of the point of springs connected to vibration body 2 can be written in the following form

$$\Phi_{k2r}=\Phi_{02}+\Gamma \Phi_{k02r}\;,\;r=1,2,3,4$$ (5)

Where **Φ**_02_ = [x_1_,y_1_]^T^, **Φ**_*k*021_ = [-*l*_*x2*_,0]^T^, **Φ**_*k*022_ = [0,*ly*_*2*_]^T^, **Φ**_*k*023_ = [*l*_*x2*_,0]^T^ and **Φ**_*k*024_ = [0,*ly*_*2*_]^T^.

The position vector of the point of springs connected to vibration body 1 can be expressed as

$$\Phi_{k1r}=\Phi_{02}+\Gamma \Phi_{k02r}-\Phi_{01}-\Gamma \Phi_{k01r}\;,\;r=1,2,3,4$$ (6)

Where **Φ**_01_ = [x_1_,y_1_]^T^, **Φ**_*k*011_ = [-*l*_*x2*_,0]^T^, **Φ**_*k*012_ = [0,*ly*_*2*_]^T^, **Φ**_*k*013_ = [*l*_*x2*_,0]^T^ and **Φ**_*k*014_ = [0,*ly*_*2*_]^T^.

The potential energy of the system can be computed as

$$V=\frac{1}{2}\sum_{i=1}^{4}{\left(\Phi_{k1r}-\Phi_{k01r}\right)}^{\text{T}}k_{1r}(\Phi_{k1r}-\Phi_{k01r})+\frac{1}{2}\sum_{r=1}^{4}{\left(\Phi_{k2r}-\Phi_{k02r}\right)}^{\text{T}}k_{2i}(\Phi_{k2r}-\Phi_{k02r})$$ (7)

The viscous dissipation function of the vibration system can be obtained

$$D=\frac{1}{2}\sum_{r=1}^{4}{\dot{\Phi }}_{k1r}{}^{\text{T}}f_{1r}{\dot{\Phi }}_{k1r}+\frac{1}{2}\sum_{r=1}^{4}{\dot{\Phi }}_{k2r}{}^{\text{T}}f_{2r}{\dot{\Phi }}_{k2r}$$ (8)

The dynamics equation of the system can be computed by the application of the Lagrange’s equation

$$\frac{d}{dt}\frac{\partial (T-V)}{\partial \dot{q}}-\frac{\partial (T-V)}{\partial q}+\frac{\partial D}{\partial \dot{q}}=Q_{i}$$ (9)

If **q** = [x_1_,x_2_,y_1_,y_2_,*ψ*_*1*,_ *ψ*_*2*,_ *φ*_*1*,_ *φ*_*2*_]^T^ is chosen as the generalized coordinates, the generalized force are: *Q*_*xi*_ = *Q*_*yi*_ = *Q*_*ψi*_ = 0, *Q*_*φi*_ = *T*_*ei*_ (*i* = 1,2). As *m*_*i*_ ⪡ *M*_*i*_ and *ψ*_*i*_⪡ 1 in the system, the inertia coupling stemming from asymmetry of the two rotors can be neglected. Substituting Eqs (4), (7) and (8) into Eq (9), we can yield the dynamic equation of the vibration system as the following form:

$$\begin{array}{l} M_{1}{\ddot{x}}_{1}-f_{x1}({\dot{x}}_{2}-{\dot{x}}_{1})-k_{x1}(x_{2}-x_{1})=m_{1}r({\ddot{\varphi }}_{1}\sin\varphi_{1}+{\dot{\varphi }}_{1}^{2}\cos\varphi_{1})\\ M_{2}{\ddot{x}}_{2}+f_{x1}({\dot{x}}_{2}-{\dot{x}}_{1})+f_{x2}{\dot{x}}_{2}+k_{x1}(x_{2}-x_{1})+k_{x2}x_{2}=m_{2}r({\ddot{\varphi }}_{2}\sin\varphi_{2}+{\dot{\varphi }}_{2}^{2}\cos\varphi_{2})\\ M_{1}{\ddot{y}}_{1}-f_{y1}({\dot{y}}_{2}-{\dot{y}}_{1})-k_{y1}(y_{2}-y_{1})=m_{1}r(-{\ddot{\varphi }}_{1}\cos\varphi_{1}+{\dot{\varphi }}_{1}^{2}\sin\varphi_{1})\\ M_{2}{\ddot{y}}_{2}+f_{y1}({\dot{y}}_{2}-{\dot{y}}_{1})+f_{y2}{\dot{y}}_{2}+k_{y1}(y_{2}-y_{1})+k_{y2}y_{2}=m_{2}r(-{\ddot{\varphi }}_{2}\cos\varphi_{2}+{\dot{\varphi }}_{2}^{2}\sin\varphi_{2})\\ J_{1}{\ddot{\psi }}_{1}-f_{\psi 1}({\dot{\psi }}_{2}-{\dot{\psi }}_{1})-k_{\psi 1}(\psi_{2}-\psi_{1})=m_{1}rl[-{\ddot{\varphi }}_{1}\cos(\varphi_{1}-\beta_{1})+{\dot{\varphi }}_{1}^{2}\sin(\varphi_{1}-\beta_{1})]\\ J_{2}{\ddot{\psi }}_{2}+f_{\psi 1}({\dot{\psi }}_{2}-{\dot{\psi }}_{1})+f_{\psi 2}{\dot{\psi }}_{2}+k_{\psi 1}(\psi_{2}-\psi_{1})+k_{\psi 2}\psi_{2}=m_{2}rl[-{\ddot{\varphi }}_{2}\cos(\varphi_{2}+\beta_{2})+{\dot{\varphi }}_{2}^{2}\sin(\varphi_{2}+\beta_{2})]\\ \left(J_{o1}+m_{1}r^{2}){\ddot{\varphi }}_{1}+f_{1}{\dot{\varphi }}_{1}=T_{e1}+m_{1}r({\ddot{x}}_{1}\sin\varphi_{1}-{\ddot{y}}_{1}\cos\varphi_{1})+m_{1}lr[{\ddot{\psi }}_{1}\cos(\varphi_{1}-\beta_{1})+{\dot{\psi }}_{1}^{2}\sin(\varphi_{1}-\beta_{1})\right]\\ \left(J_{o2}+m_{2}r^{2}){\ddot{\varphi }}_{2}+f_{2}{\dot{\varphi }}_{2}=T_{e2}+m_{2}r({\ddot{x}}_{2}\sin\varphi_{2}-{\ddot{y}}_{2}\cos\varphi_{2})+m_{2}lr[{\ddot{\psi }}_{2}\cos(\varphi_{2}+\beta_{2})+{\dot{\psi }}_{2}^{2}\sin(\varphi_{2}+\beta_{2})\right] \end{array}$$ (10)

## Method description

### Coupling characteristics between the two vibro-bodies

As shown in Fig 1, the phase angular of the rotors are defined as follows:

$$\varphi_{1}=\varphi +\alpha ,\varphi_{2}=\varphi -\alpha$$ (11)

Assuming that the average value of the angular velocity of the two exciters over time is *ω*_*m*_, and the instantaneous change coefficients of $\dot{\varphi }$ and $\dot{\alpha }$ are *ε*_1_ and *ε*_2_ (i.e.$\dot{\varphi }=(1+\varepsilon_{1})\omega_{m}$,$\dot{\alpha }=\varepsilon_{2}\omega_{m}$) when the two unbalanced rotors operate in the synchronous state, respectively. So, the velocity of the phase angular can be expressed as [22]

$$\begin{array}{l} {\dot{\varphi }}_{1}=(1+\varepsilon_{1}+\varepsilon_{2})\omega_{m},\\ {\dot{\varphi }}_{2}=(1+\varepsilon_{1}+\varepsilon_{2})\omega_{m}. \end{array}$$ (12)

Moreover, the accelerations of the phase angular can be written as

$$\begin{array}{l} {\ddot{\varphi }}_{1}=({\dot{\varepsilon }}_{1}+{\dot{\varepsilon }}_{2})\omega_{m},\\ {\ddot{\varphi }}_{2}=({\dot{\varepsilon }}_{1}-{\dot{\varepsilon }}_{2})\omega_{m}. \end{array}$$ (13)

Because the motion and the load torque of the vibration system are periodical, the angular velocities of the two rotors change periodically. If the two rotors excited by two induction motors operating synchronously, the average values of their instantaneous change coefficients of the angular velocities and the angular accelerations over one period must be zero, i.e., ${\bar{\varepsilon }}_{1}=0$,${\bar{\varepsilon }}_{2}=0$, ${\dot{\bar{\varepsilon }}}_{1}=0$ and ${\dot{\bar{\varepsilon }}}_{2}=0$. In the case, the change of angular velocities of the two unbalanced rotors has little influence on the responses of the whole system. According to Eq (10), the first six equations of the equation are coupling equations with Multi-DOF. To obtain their steady response solutions we can use the Laplace transformation method to the equations considering the initial conditions. The steady responses of the DOFs can be expressed as

$$\begin{array}{l} x_{1}=rr_{m}\mu_{x11}\cos(\varphi +\alpha -r_{x11})+\eta rr_{m}\mu_{x12}\cos(\varphi -\alpha -r_{x12}),\\ x_{2}=\eta rr_{m}\mu_{x21}\cos(\varphi -\alpha -r_{x21})+rr_{m}\mu_{x22}\cos(\varphi +\alpha -r_{x22}),\\ y_{1}=rr_{m}\mu_{y11}\sin(\varphi +\alpha -r_{y11})+\eta rr_{m}\mu_{y12}\sin\varphi -\alpha -r_{y12}),\\ y_{2}=\eta rr_{m}\mu_{y21}sin(\varphi -\alpha -r_{y21})+rr_{m}\mu_{y22}sin(\varphi +\alpha -r_{y22}),\\ \psi_{1}=\frac{r_{m}r_{l}r\mu_{\psi 11}}{l}\sin(\varphi +\alpha -\beta_{1}-r_{\psi 11})+\frac{\eta r_{m}r_{l}r\mu_{\psi 12}}{l}\sin(\varphi -\alpha -\beta_{2}-r_{\psi 12}),\\ \psi_{2}=\frac{\eta r_{m}r_{l}r\mu_{\psi 21}}{l}sin(\varphi -\alpha -\beta_{2}-r_{\psi 21})+\frac{r_{m}r_{l}r\mu_{\psi 22}}{l}sin(\varphi +\alpha -\beta_{1}-r_{\psi 22}). \end{array}$$ (14)

In Eq (14), *μ*_x11_, *μ*_x12_, *μ*_x21_ and *μ*_x22_ represent the coupling (transfer) coefficients in the *x*-direction; *μ*_*y*11_, *μ*_y12_, *μ*_y21_ and *μ*_y22_ represent the coupling coefficients in the *y*-direction; *μ*_*ψ*11_, *μ*_*ψ*12_, *μ*_*ψ*21_ and *μ*_*ψ*22_ represent the coupling coefficients in the *ψ*-direction. These coupling coefficients and mathematical symbols in Eq (14) are listed Appendix A in S1 File, by which we can estimate the value of the coupling coefficients between the two vibro-bodies. They are dependent on the stiffness coefficients and the damping coefficients of the springs. The stronger ability of the coupling between the bodies, the larger the value of the coupling coefficients is.

**Fig 2:**
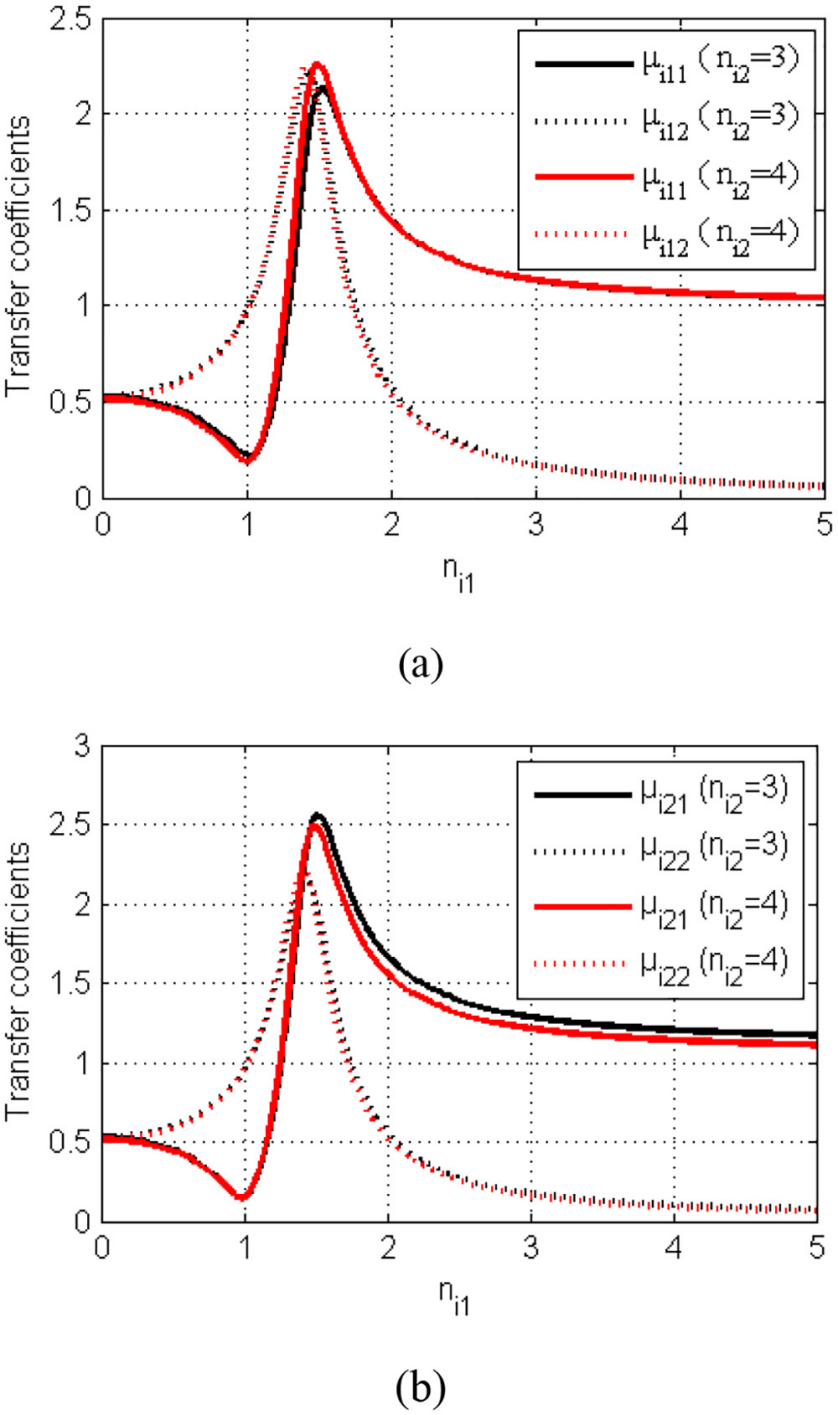
The coupling coefficients between the two vibration bodies for ξ_i_1 = *ξ*_*i2*_ = 0.075.

Fig 2 describes the relation of the coupling coefficients between two vibration bodies for the different value of *n*_*i*1_ and *n*_*i*2_. Table 1 shows the values of the transfer coefficients of some special points in Fig 2. It can be seen that *μ*_*i*11_ ≈ *μ*_*i*21_ and *μ*_*i*12_ ≈ *μ*_*i*22_ when the value of parameters *n*_*i*2_ is different. The frequency ratio *n*_*i*1_ in region *n*_*i*1_ ∈ (0,1) is proportional to parameters *μ*_*i*12_ and *μ*_*i*22_, and inversely proportional *μ*_*i*11_ and *μ*_*i*21_. The frequency ratio *n*_*i*1_ in region *n*_*i*1_ ∈ (1,1.5) is proportional to parameters *μ*_*i*11_, *μ*_*i*12_, *μ*_*i*21_ and *μ*_*i*22_. The frequency ratio *n*_*i*1_ in region *n*_*i*1_ ∈ (1.5,5)is proportional to parameters *μ*_*i*11_ and *μ*_*i*21_, and inversely proportional to parameters *μ*_*i*12_ and *μ*_*i*22_. According to the value of the coupling coefficients we can define the coupling type of the vibration system. Obviously, three kinds of the coupling type can be described in following:

Case 1: the near-resonance system coupled with the far-resonance system (NVS-FVS) considering *n*_*i*1_ ∈ (0,1) and *n*_*i2*_ = 4.
Case 2: the resonance system coupled with the far-resonance vibration system (RVS-FVS) considering *n*_*i*1_ ∈ (1,2) and *n*_*i2*_ = 4.
Case 3: the far-resonance system coupled with the far-resonance system (FVS-FVS) considering *n*_*i*1_ ∈ (2,5) and *n*_*i2*_ = 4.

**Table 1:** The values of the transfer coefficients for *ξ*_i_1 = *ξ*_*i2*_ = 0.075.

*n*_*i1*_ = 3	*n*_*i2*_	0.5	1.0	1.5	2.0	3.0	4.0	5.0
	*μ*_*i*11_	0.46	0.22	2.12	1.44	1.13	1.07	1.04
	*μ*_*i*12_	0.58	0.96	2.06	0.58	0.18	0.09	0.06
	*μ*_*i*21_	0.45	0.14	2.53	1.67	1.28	1.25	1.17
	*μ*_*i*22_	0.59	0.96	2.0	0.56	0.19	0.10	0.08
*n*_*i1*_ = 4	*μ*_*i*11_	0.46	0.22	2.25	1.44	1.13	1.07	1.04
	*μ*_*i*12_	0.58	0.96	2.01	0.54	0.17	0.09	0.06
	*μ*_*i*21_	0.44	0.14	2.49	1.56	1.22	1.14	1.11
	*μ*_*i*22_	0.58	0.96	1.97	0.52	0.18	0.09	0.07

In this paper, rigid vibro-body 1 is connected with vibro-body 2 by the stronger stiffness springs, and vibro-body 1 is connected a fix foundation with the weaker stiffness spring. Therefore, in the following theoretical analysis, we will chose case 1 to discuss the synchronization and stability of the system, and the value of the coupling coefficients should be less than or equal to 1.

### Coupling characteristics between the two rotors

Differentiating the six formulas in Eq (14) with respect to time *t* by the chain rule, we can obtain ${\ddot{x}}_{1},{\ddot{x}}_{2},{\ddot{y}}_{1},{\ddot{y}}_{2},{\ddot{\psi }}_{1}$ and ${\ddot{\psi }}_{2}$ with neglecting the terms larger than one order of parameters *ε*_1_ and *ε*_2_. Substituting them into the last two formulas of Eq (10) and integrating them over *φ* = 2*π*, respectively, we can obtain:

$$\begin{array}{l} J_{01}\omega_{m}({\dot{\bar{\varepsilon }}}_{1}+{\dot{\bar{\varepsilon }}}_{2})+f_{1}\omega_{m}(1+{\bar{\varepsilon }}_{1}+{\bar{\varepsilon }}_{2})+m_{1}r^{2}\omega_{m}{\bar{T}}_{L1}={\bar{T}}_{e1},\\ J_{02}\omega_{m}({\dot{\bar{\varepsilon }}}_{1}-{\dot{\bar{\varepsilon }}}_{2})+f_{1}\omega_{m}(1+{\bar{\varepsilon }}_{1}-{\bar{\varepsilon }}_{2})+m_{1}r^{2}\omega_{m}{\bar{T}}_{L2}={\bar{T}}_{e2}. \end{array}$$ (15)

with

$$\begin{array}{l} {\bar{T}}_{L1}={\chi^{\prime }}_{11}({\dot{\bar{\varepsilon }}}_{1}+{\dot{\bar{\varepsilon }}}_{2})+{\chi^{\prime }}_{12}({\dot{\bar{\varepsilon }}}_{1}-{\dot{\bar{\varepsilon }}}_{2})+\omega_{m}[({\bar{\varepsilon }}_{1}+{\bar{\varepsilon }}_{2})\chi_{11}+({\bar{\varepsilon }}_{1}-{\bar{\varepsilon }}_{2})\chi_{12}+\chi_{a1}+\chi_{f1}],\\ {\bar{T}}_{L2}={\chi^{\prime }}_{21}({\dot{\bar{\varepsilon }}}_{1}+{\dot{\bar{\varepsilon }}}_{2})+{\chi^{\prime }}_{22}({\dot{\bar{\varepsilon }}}_{1}-{\dot{\bar{\varepsilon }}}_{2})+\omega_{m}[({\bar{\varepsilon }}_{1}+{\bar{\varepsilon }}_{2})\chi_{21}+({\bar{\varepsilon }}_{1}-{\bar{\varepsilon }}_{2})\chi_{22}+\chi_{a2}+\chi_{f2}]. \end{array}$$ (16)

where

$$\begin{array}{l} {\chi^{\prime }}_{11}=\frac{1}{2}m_{1}r\omega^{2}W_{c1},\;\;\;\;\;\;\;\;\;\;{\chi^{\prime }}_{12}=\frac{1}{2}m_{1}r\omega^{2}[{W^{\prime }}_{c1}\cos(2\alpha +\theta_{c1})-{W^{\prime }}_{s1}sin(2\alpha +\theta_{s1})],\\ \chi_{11}=m_{1}r\omega^{2}W_{s1},\;\;\;\;\;\;\;\;\;\;\;\;\;\;\;\;\chi_{12}=m_{1}r\omega^{2}[{W^{\prime }}_{c1}sin(2\alpha +\theta_{c1})+{W^{\prime }}_{s1}\cos(2\alpha +\theta_{s1})],\\ \chi_{a1}=\frac{1}{2}m_{1}r\omega^{2}[{W^{\prime }}_{c1}sin(2\alpha +\theta_{c1})],\;\;\;\;\;\;\;\;\chi_{f1}=\frac{1}{2}m_{1}r\omega^{2}[{W^{\prime }}_{s1}\cos(2\alpha +\theta_{s1})+{W^{\prime }}_{s1}],\\ {\chi^{\prime }}_{21}=\frac{1}{2}m_{1}r\omega^{2}[{W^{\prime }}_{c2}\cos(2\alpha +\theta_{c2})+{W^{\prime }}_{s2}\sin(2\alpha +\theta_{s2})],\;\;\;\;\;\;\;\;\;\;{\chi^{\prime }}_{22}=\frac{1}{2}m_{1}r\omega^{2}W_{c2},\\ \chi_{21}=m_{1}r\omega^{2}[-{W^{\prime }}_{c2}\sin(2\alpha +\theta_{c2})+{W^{\prime }}_{s2}\cos(2\alpha +\theta_{s2})],\;\;\;\;\;\;\;\;\;\;\chi_{21}=m_{1}r\omega^{2}W_{s2},\\ \chi_{a2}=\frac{1}{2}m_{1}r\omega^{2}[-{W^{\prime }}_{c2}\sin(2\alpha +\theta_{c2})],\;\;\;\;\;\;\;\;\;\;\;\;\;\chi_{f2}=\frac{1}{2}m_{1}r\omega^{2}[{W^{\prime }}_{s2}\cos(2\alpha +\theta_{s2})-W_{s2}]. \end{array}$$

Parameters *W*_*c*1_, *W*_*c2*_, ${W^{\prime }}_{c1}$, ${W^{\prime }}_{c2}$, *W*_*s*1_, *W*_*s*2_, ${W^{\prime }}_{s1}$ and ${W^{\prime }}_{s2}$ can be found Appendix B in S1 File. Compared with the change value of angular velocities of the two rotors, that of *α*, *ε*_1_, *ε*_2_, ${\dot{\varepsilon }}_{1}$ and ${\dot{\varepsilon }}_{2}$ can be considered small parameters (i.e., *ε*_1_ ⪡ 1, *ε*_2_ ⪡ 1, ${\dot{\varepsilon }}_{1}\ll 1$ and ${\dot{\varepsilon }}_{2}\ll 1$). During the above-mentioned integration, the value of parameters *α*, *ε*_1_, *ε*_2_, ${\dot{\varepsilon }}_{1}$ and ${\dot{\varepsilon }}_{2}$ are assumed to be the average values of their integration $\bar{\alpha }$,${\bar{\varepsilon }}_{1}$, ${\bar{\varepsilon }}_{2}$, ${\dot{\bar{\varepsilon }}}_{1}$ and ${\dot{\bar{\varepsilon }}}_{2}$, respectively [23]. In addition, compared with parameters *W*_*c*1_, *W*_*c2*_, ${W^{\prime }}_{c1}$ and ${W^{\prime }}_{c2}$, the other parameters *W*_*s*1_, *W*_*s*2_, ${W^{\prime }}_{s1}$ and ${W^{\prime }}_{s2}$ are very small in the vibration system as the value of the damping ratio coefficients is very small. Hence parameters *W*_*s*1_, *W*_*s*2_, ${W^{\prime }}_{s1}$ and ${W^{\prime }}_{s2}$ can be ignored in the in the expressions of ${\chi^{\prime }}_{12}$, *χ*_12_, ${\chi^{\prime }}_{21}$ and *χ*_21_.

According to Ref. [22], in Eq (15) ${\bar{T}}_{e1}$ and ${\bar{T}}_{e2}$ can be expressed as

$$\begin{array}{l} {\bar{T}}_{e1}=T_{e01}-k_{e1}({\bar{\varepsilon }}_{1}+{\bar{\varepsilon }}_{2}),\\ {\bar{T}}_{e2}=T_{e02}-k_{e2}({\bar{\varepsilon }}_{1}-{\bar{\varepsilon }}_{2}). \end{array}$$ (17)

Where *T*_*e*01_ and *T*_*e*02_ are the electromagnetic torques of the two induction motors rotating at the angular velocity of *ω*_*m*_, *k*_*e*1_ and *k*_*e2*_ are the angular stiffness of the two motors.

Firstly adding the two formulas of Eq (15) as the first row, then subtracting the two formulas of Eq (15) as the second row, next substituting Eqs (16) and (17) into Eq (15), introducing the following non-dimensional parameters *ρ*_*1*_ = 1+*W*_*c*1_/2, *ρ*_*2*_ = *η*+*W*_*c2*_/2, $\kappa_{1}=k_{e1}/m_{1}r^{2}\omega_{m}^{2}+f_{1}\omega_{m}/m_{1}r^{2}\omega_{m}^{2}+W_{s1}$ and $\kappa_{2}=k_{e2}/m_{1}r^{2}\omega_{m}^{2}+f_{2}\omega_{m}/m_{1}r^{2}\omega_{m}^{2}+W_{s2}$ into the above-mention equations, Eq (15) can be rewritten as the matrix form:

$$A\dot{\varepsilon }=B\varepsilon +C.$$ (18)

where

$$\begin{array}{l} A=\left[\begin{array}{cc} \rho_{1}+\rho_{2}+{W^{\prime }}_{c1}\cos(2\alpha +\theta_{c1})/2+{W^{\prime }}_{c2}\cos(2\alpha +\theta_{c2})/2 & \rho_{1}-\rho_{2}-{W^{\prime }}_{c1}\cos(2\alpha +\theta_{c1})/2+{W^{\prime }}_{c2}\cos(2\alpha +\theta_{c2})/2\\ \rho_{1}-\rho_{2}-{W^{\prime }}_{c1}\cos(2\alpha +\theta_{c1})/2+{W^{\prime }}_{c2}\cos(2\alpha +\theta_{c2})/2 & \rho_{1}+\rho_{2}-{W^{\prime }}_{c1}\cos(2\alpha +\theta_{c1})/2-{W^{\prime }}_{c2}\cos(2\alpha +\theta_{c2})/2 \end{array}\right],\\ B=\left|\begin{array}{cc} \kappa_{1}+\kappa_{2}+{W^{\prime }}_{c1}\sin(2\alpha +\theta_{c1})-{W^{\prime }}_{c2}sin(2\alpha +\theta_{c2}) & \kappa_{1}-\kappa_{2}-{W^{\prime }}_{c1}\sin(2\alpha +\theta_{c1})-{W^{\prime }}_{c2}sin(2\alpha +\theta_{c2})\\ \kappa_{1}-\kappa_{2}+{W^{\prime }}_{c1}\sin(2\alpha +\theta_{c1})+{W^{\prime }}_{c2}sin(2\alpha +\theta_{c2}) & \kappa_{1}+\kappa_{2}-{W^{\prime }}_{c1}\sin(2\alpha +\theta_{c1})+{W^{\prime }}_{c2}sin(2\alpha +\theta_{c2}) \end{array}\right|,\;\varepsilon =\left|\begin{array}{l} \varepsilon_{1}\\ \varepsilon_{2} \end{array}\right|,\\ C=\left|\begin{array}{c} T_{e01}+T_{e2}-f_{1}\omega_{m}-f_{2}\omega_{m}=\frac{1}{2}m_{1}r^{2}\omega_{m}^{2}[{W^{\prime }}_{c1}sin(2\alpha +\theta_{c1})-{W^{\prime }}_{c2}\sin(2\alpha +\theta_{c2})+{W^{\prime }}_{s1}\cos(2\alpha +\theta_{s1})+{W^{\prime }}_{s2}\cos(2\alpha +\theta_{s2})+W_{s1}+W_{s2}]\\ T_{e01}-T_{e2}-f_{1}\omega_{m}+f_{2}\omega_{m}=\frac{1}{2}m_{1}r^{2}\omega_{m}^{2}[{W^{\prime }}_{c1}sin(2\alpha +\theta_{c1})+{W^{\prime }}_{c2}\sin(2\alpha +\theta_{c2})+{W^{\prime }}_{s1}\cos(2\alpha +\theta_{s1})-{W^{\prime }}_{s2}\cos(2\alpha +\theta_{s2})+W_{s1}-W_{s2}] \end{array}\right| \end{array}$$

In Eq (18), symbol **A** is considered as the inertia moments of the two unbalanced rotors and defined as the inertia coupling matrix. Symbol **B** is considered as the stiffness of angular velocities of the two unbalanced rotors and defined as the stiffness matrix. Symbol **C** is the matrix related to the electromagnetic torque and the load torque of the induction motors. Therefore, eq (18), the non-dimensional coupling equation, describes the dynamic coupling characteristics of the two unbalanced rotors.

### Condition of implementing synchronization

When the two rotors synchronously rotate in the vibration system, we have $\dot{\bar{\varepsilon }}=0$,$\bar{\varepsilon }=0$ and **c** = 0. According to the above analysis for the coupling characteristics between the two vibro-bodies (*μ*_*i*11_ ≈ *μ*_*i*21_, *μ*_*i*12_ ≈ *μ*_*i*22_ *μ*_*i*12_ ≈ *μ*_*i*11_and *μ*_*i*22_ > *μ*_*i*21_ in NVS-FVS), we can consider that ${W^{\prime }}_{c1}\approx {W^{\prime }}_{c2}\approx {W^{\prime }}_{c}$,${W^{\prime }}_{s1}\approx {W^{\prime }}_{s2}\approx {W^{\prime }}_{s}$, *θ*_*c*1_ ≈ *θ*_*c*2_ ≈ *θ*_*c*_ and *θ*_*s*1_ ≈ *θ*_*s*2_ ≈ *θ*_*s*_ according to Appendix B in S1 File. Moreover, we assume that the values of the damping coefficient of the springs is equal each other because of the identical and small damping of the springs in NVS-FVS. Thus, we have *r*_*x*11_ ≈ *r*_*x*12_, *r*_*y*11_ ≈ *r*_*y*12_ and *r*_*ψ*11_ ≈ *r*_*ψ*12_. Substituting them into Eq (18) and rearranging them, we have

$$T_{e01}-f_{1}\omega_{m}+T_{e2}-f_{2}\omega_{m}=m_{1}r^{2}\omega_{m}^{2}{W^{\prime }}_{s}\cos(2\bar{\alpha }+\theta_{s})+\frac{1}{2}m_{1}r^{2}\omega_{m}^{2}(W_{s1}+W_{s2})\;,$$ (19)

$$T_{e01}-f_{1}\omega_{m}-(T_{e2}-f_{2}\omega_{m})=m_{1}r^{2}\omega_{m}^{2}{W^{\prime }}_{c}sin(2\bar{\alpha }+\theta_{c})+\frac{1}{2}m_{1}r^{2}\omega_{m}^{2}(W_{s1}-W_{s2})$$ (20)

Eq (19) is the equation of torque balance of the vibration system in the synchronous state, which serves to find the approximation of angular velocity *ω*_*m*_. Moreover, the second formula of Eq (20) is difference equation of the balanced torque of the two rotors in the synchronous state, which serves to determine the approximation of stable phase difference 2*α*.

Rewriting Eq (20), we obtain

$$2\bar{\alpha }=\arcsin(T_{D}/T_{C})-\theta_{c}\;,$$ (21)

where

$$\begin{array}{l} T_{D}=T_{R1}-T_{R2},\\ T_{R1}=T_{e01}-f_{1}\omega_{m}-m_{1}r^{2}\omega_{m}^{2}W_{s1}/2,\\ T_{R2}=T_{e02}-f_{2}\omega_{m}-m_{1}r^{2}\omega_{m}^{2}W_{s2}/2,\\ T_{C}=m_{1}r^{2}\omega_{m}^{2}{W^{\prime }}_{c}. \end{array}$$

*T*_*C*_ is the torque of synchronization capture; *T*_*D*_ is the difference between the residual electromagnetic torques of the two induction motors; *T*_*R1*_ and *T*_*R2*_ are the residual electromagnetic of induction motor 1 and 2, respectively.

Since $\left|sin2\bar{\alpha }+\theta_{c}\right|\leq 1$, the synchronization condition of the vibration system can be expressed as

$$T_{C}\geq \left|T_{D}\right|$$ (22)

It is indicated that the torque of synchronization capture should be equal or greater than the absolute value of the difference between the residual electromagnetic torques of the two induction motors for the synchronization implementation of the system.

### Synchronization stability of the rotors

If the system parameters satisfy the condition of implementing synchronization, the stable phase difference and the synchronous angular velocity of the rotors can be solved with the numerical method. In addition, the stability region of the phase difference can be confirmed with the Lyapunov theory. When **C** = 0, Eq (18) is the generalized system:

$$A\dot{\varepsilon }=B\varepsilon$$ (23)

Linearizing Eq (23) around $\bar{\alpha }={\bar{\alpha }}_{0}$ with the Taylor expansion, and appending $\Delta \dot{\alpha }=\omega_{m}^{*}{\bar{\varepsilon }}_{2}$ as the third row, the first-order approximate linear equation of the two unbalanced rotors can be obtained

$$\dot{\varsigma }=Z\varsigma \;,$$ (24)

with

$$Z=-A_{0}{}^{-1}B_{0}\;,$$ (25)

and

$$\begin{array}{l} A_{0}=\left|\begin{array}{ccc} \rho_{1}+\rho_{2}+{W^{\prime }}_{c}\cos(2{\bar{\alpha }}_{0}+\theta_{c})/2 & \rho_{1}-\rho_{2} & 0\\ \rho_{1}-\rho_{2} & \rho_{1}+\rho_{2}-{W^{\prime }}_{c}\cos(2{\bar{\alpha }}_{0}+\theta_{c}) & 0\\ 0 & 0 & 1 \end{array}\right|,\;\varsigma =\left|\begin{array}{c} \varepsilon_{1}\\ \begin{array}{l} \varepsilon_{2}\\ \Delta \alpha \end{array} \end{array}\right|,\\ B_{0}=\left|\begin{array}{ccc} \kappa_{1}+\kappa_{2} & \kappa_{1}-\kappa_{2}-2{W^{\prime }}_{c}\sin(2{\bar{\alpha }}_{0}+\theta_{c}) & 2\omega_{m}^{*}{W^{\prime }}_{\text{s}}\sin(2{\bar{\alpha }}_{0}+\theta_{s})\\ \kappa_{1}-\kappa_{2}+2{W^{\prime }}_{c}\sin(2{\bar{\alpha }}_{0}+\theta_{c}) & \kappa_{1}+\kappa_{2} & -2\omega_{m}^{*}{W^{\prime }}_{c}\cos2(2{\bar{\alpha }}_{0}+\theta_{c})\\ 0 & \omega_{m}^{*} & 0 \end{array}\right|. \end{array}$$

where $\Delta \alpha =\bar{\alpha }-\alpha_{0}$. It should be noted that matrices **A**_0_ and **B**_0_ represent the linearization matrices **A** and **B** for $\bar{\alpha }=\alpha_{0}$ and $\omega_{m}=\omega_{m}^{*}$, respectively. Meanwhile, matrices **A**_0_ and **B**_0_ are the simplification style of matrices **A** and **B** considering *μ*_*i*11_ ≈ *μ*_*i*21_, *μ*_*i*12_ ≈ *μ*_*i*22_,${W^{\prime }}_{c1}\approx {W^{\prime }}_{c2}\approx {W^{\prime }}_{c}$, ${W^{\prime }}_{s1}\approx {W^{\prime }}_{s2}\approx {W^{\prime }}_{s}$, *θ*_*c*1_ ≈ *θ*_*c*2_ ≈ *θ*_*c*_ and *θ*_*s*1_ ≈ *θ*_*s*2_ ≈ *θ*_*s*_, and *r*_*i*11_ ≈ *r*_*i*12_ ≈ *r*_*i*21_ ≈ *r*_*i*22_.

Exponential time-dependence of the form **ς** = **u**(*λt*) is now assumed. Inserting it into Eq (24), and solving the determinant equation |**Z**−*λ* **I**| = 0, we can yield the characteristic equation

$$a_{0}\lambda^{3}+a_{1}\lambda^{2}+a_{2}\lambda +a_{3}=0$$ (26)

where a_0_ = 1,$a_{1}=\omega_{m}^{*}H_{1}/H_{0}$, $a_{2}=\omega_{m}^{*2}H_{2}/H_{0}$ and $a_{3}=\omega_{m}^{*3}H_{2}/H_{0}$.

$$\begin{array}{c} H_{0}=4\rho_{1}\rho_{2}-{W^{\prime }}_{c}^{2}\cos^{2}(2{\bar{\alpha }}_{0}+\theta_{c})+{W^{\prime }}_{s}^{2}\sin^{2}(2{\bar{\alpha }}_{0}+\theta_{s}),\\ H_{1}=[-2{W^{\prime }}_{s}(\rho_{1}+\rho_{2})\cos(2{\bar{\alpha }}_{0}+\theta_{s})+2{W^{\prime }}_{s}{W^{\prime }}_{c}\cos(2{\bar{\alpha }}_{0}+\theta_{s})cos(2{\bar{\alpha }}_{0}+\theta_{c})]/\omega_{m}^{*}\\ +(4\kappa_{1}\rho_{2}+4\kappa_{2}\rho_{1})+2{W^{\prime }}_{c}{W^{\prime }}_{s}\cos(2{\bar{\alpha }}_{0}+\theta_{s})cos(2{\bar{\alpha }}_{0}+\theta_{c})\\ +4{W^{\prime }}_{c}{W^{\prime }}_{s}\omega_{m0}^{*}\sin(2{\bar{\alpha }}_{0}+\theta_{s})sin(2{\bar{\alpha }}_{0}+\theta_{c})-2{W^{\prime }}_{s}(\rho_{1}+\rho_{2})\cos(2{\bar{\alpha }}_{0}+\theta_{s}),\\ H_{2}=4\kappa_{1}\kappa_{2}+2{W^{\prime }}_{c}^{2}+2{W^{\prime }}_{c}^{2}sin^{2}(2{\bar{\alpha }}_{0}+\theta_{c})-2{W^{\prime }}_{s}^{2}\sin^{2}(2{\bar{\alpha }}_{0}+\theta_{s})+2{W^{\prime }}_{s}(\kappa_{1}+\kappa_{2})\cos(2{\bar{\alpha }}_{0}+\theta_{s})\\ +2{W^{\prime }}_{c}(\rho_{1}+\rho_{2})\cos(2{\bar{\alpha }}_{0}+\theta_{c})+2{W^{\prime }}_{s}(\rho_{1}-\rho_{2})sin(2{\bar{\alpha }}_{0}+\theta_{s})\\ -[4{W^{\prime }}_{s}^{2}\cos^{2}(2{\bar{\alpha }}_{0}+\theta_{s})+2{W^{\prime }}_{s}(\kappa_{1}+\kappa_{2})\cos(2{\bar{\alpha }}_{0}+\theta_{s})]/\omega_{m}^{*},\\ H_{3}=2{W^{\prime }}_{c}(\kappa_{1}+\kappa_{2})\cos(2{\bar{\alpha }}_{0}+\theta_{c})+2{W^{\prime }}_{s}(\kappa_{1}-\kappa_{2})\sin(2{\bar{\alpha }}_{0}+\theta_{s})+4{W^{\prime }}_{c}{W^{\prime }}_{s}. \end{array}$$

In the vibration system, the value of parameter ${W^{\prime }}_{c}$ is far larger than $W_{s}{}^{\prime }$ because the value of the damping ratio (*ξ*_*ni*_ < 0.05)is very small [14]. In the following calculation, we will ignore parameter $W_{s}{}^{\prime }$ to simplify *H*_0_, *H*_1_, *H*_2_ and *H*_3_ as

$$\begin{array}{c} {\tilde{H}}_{0}=4\rho_{1}\rho_{2}-{W^{\prime }}_{c}^{2}\cos^{2}(2{\bar{\alpha }}_{0}+\theta_{c}),\\ {\tilde{H}}_{1}=4\kappa_{1}\rho_{2}+4\kappa_{2}\rho_{1},\\ {\tilde{H}}_{2}=4\kappa_{1}\kappa_{2}+2{W^{\prime }}_{c}^{2}+2{W^{\prime }}_{c}^{2}\sin^{2}(2{\bar{\alpha }}_{0}+\theta_{c})+2{W^{\prime }}_{c}(\rho_{1}+\rho_{2})\cos(2{\bar{\alpha }}_{0}+\theta_{c}),\\ {\tilde{H}}_{3}=2{W^{\prime }}_{c}(\kappa_{1}+\kappa_{2})\cos(2{\bar{\alpha }}_{0}+\theta_{c}). \end{array}$$ (27)

If all the roots of Eq (26) have negative real parts, the phase difference of two unbalance rotors is asymptotically stable. According to the Routh-Hurwitz criterion, the asymptotic stability condition of the synchronization state of the two rotors is deduced

$$a_{0}>0\;,\;\;a_{1}\;or\;a_{2}>0\;,\;a_{3}>0\;,\;a_{1}a_{2}-a_{0}a_{3}>0$$ (28)

Base on Eqs (27) and (28), we can employ the two following hypotheses to discuss the stability region of the synchronization state of the two unbalanced rotors.

Hypothesis (1): If ${\tilde{H}}_{0}>0$, only conditions ${\tilde{H}}_{1}$ or ${\tilde{H}}_{2}>0$,${\tilde{H}}_{3}>0$ and ${\tilde{H}}_{1}{\tilde{H}}_{2}-a_{0}{\tilde{H}}_{0}{\tilde{H}}_{3}>0$ satisfied, the asymptotic stability of the synchronization state of the rotors would be implemented.

By ${\tilde{H}}_{0}>0$,${\tilde{H}}_{1}>0$, *κ*_1_ > 0 and *κ*_2_ > 0,we have

$$\rho_{1}>0,\rho_{2}>0\;,\;\;4\rho_{1}\rho_{2}-{W^{\prime }}_{c}^{2}\cos(2{\bar{\alpha }}_{0}+\theta_{c})>0$$ (29)

In addition, by ${\tilde{H}}_{3}>0$ we obtain

$${W^{\prime }}_{c}\cos(2{\bar{\alpha }}_{0}+\theta_{c})>0$$ (30)

Substituting ${\tilde{H}}_{0},{\tilde{H}}_{1},{\tilde{H}}_{2}$ and ${\tilde{H}}_{3}$ into ${\tilde{H}}_{1}{\tilde{H}}_{2}-a_{0}{\tilde{H}}_{0}{\tilde{H}}_{3}$, which can be written as

$$\begin{array}{c} {\tilde{H}}_{1}{\tilde{H}}_{2}-a_{0}{\tilde{H}}_{0}{\tilde{H}}_{3}=8(\kappa_{1}\rho_{2}+\kappa_{2}\rho_{1})[{W^{\prime }}_{c}^{2}\sin^{2}(2{\bar{\alpha }}_{0}+\theta_{c})+{W^{\prime }}_{c}^{2}+2\kappa_{1}\kappa_{2}]\\ +8(\kappa_{1}\rho_{2}^{2}+\kappa_{2}\rho_{1}^{2}){W^{\prime }}_{c}\cos(2{\bar{\alpha }}_{0}+\theta_{c})+2(\kappa_{1}+\kappa_{2}){W^{\prime }}_{c}^{3}\cos^{3}(2{\bar{\alpha }}_{0}+\theta_{c}) \end{array}$$ (31)

Obviously, when *κ*_1_ > 0, *κ*_2_ > 0, *ρ*_1_ > 0, *ρ*_2_ > 0 and $4\rho_{1}\rho_{2}-{W^{\prime }}_{c}^{2}\cos2\alpha_{0}>0$, we have ${\tilde{H}}_{1}{\tilde{H}}_{2}-a_{0}{\tilde{H}}_{0}{\tilde{H}}_{3}>0$, and so the asymptotic stability of the synchronization state of the two rotors can be carried out. In the light of parameter ${W^{\prime }}_{c}\approx {W^{\prime }}_{c1}\approx {W^{\prime }}_{c2}$ larger than zero in Appendix B in S1 File, we have $\cos(2{\bar{\alpha }}_{0}+\theta_{c})>0$ according to Eq (29). Thus, one can see that $2{\bar{\alpha }}_{0}+\theta_{c}\in (-\pi /2,\pi /2)$, from which the region of the stable phase difference is confirmed by parameter *θ*_*c*_ (i.e., *θ*_*c*1_ or *θ*_*c*2_ in Appendix B in S1 File).

Hypothesis (2): If ${\tilde{H}}_{0}<0$, only conditions ${\tilde{H}}_{1}$ or ${\tilde{H}}_{2}<0$,${\tilde{H}}_{3}<0$, ${\tilde{H}}_{1}{\tilde{H}}_{2}-a_{0}{\tilde{H}}_{0}{\tilde{H}}_{3}>0$ are satisfied, the asymptotic stability of the synchronization state of the rotors would be implemented.

By ${\tilde{H}}_{0}<0$, we have $4\rho_{1}\rho_{2}-{W^{\prime }}_{c}^{2}\cos^{2}(2{\bar{\alpha }}_{0}+\theta_{c})<0$. Moreover, by ${\tilde{H}}_{1}<0$, and we obtain *κ*_1_ < 0, *κ*_2_ < 0, *β*_1_ > 0, *β*_2_ > 0 or *κ*_1_ > 0, *κ*_2_ > 0, *ρ*_1_ < 0, *ρ*_2_ < 0. Then by ${\tilde{H}}_{3}<0$, and we acquire ${W^{\prime }}_{c}\cos(2{\bar{\alpha }}_{0}+\theta_{c})<0$.

Obviously, ${\tilde{H}}_{1}{\tilde{H}}_{2}-a_{0}{\tilde{H}}_{0}{\tilde{H}}_{3}$ is less than zero according to Eq (31) when ${\tilde{H}}_{0}<0$, ${\tilde{H}}_{1}$ or ${\tilde{H}}_{2}<0$,${\tilde{H}}_{3}<0$. Therefore, this is not in accordance with stability condition of hypotheses (2).

### Numerical Discussions

Above-mentioned sections have given some theoretical discussions in the simplified form on synchronization problem for the near-resonance vibration system coupled with the far-resonance vibration system. This section will quantitatively discuss the numerical results of the stable phase difference.

Base on the balance of the force moment, the value of *r*_*l*_ can be written as

$$r_{l\max}^{2}=\underset{l\to \infty }{\lim}r_{l}^{2}=\frac{1}{\eta r_{m}}+1$$ (32)

**Fig 3:**
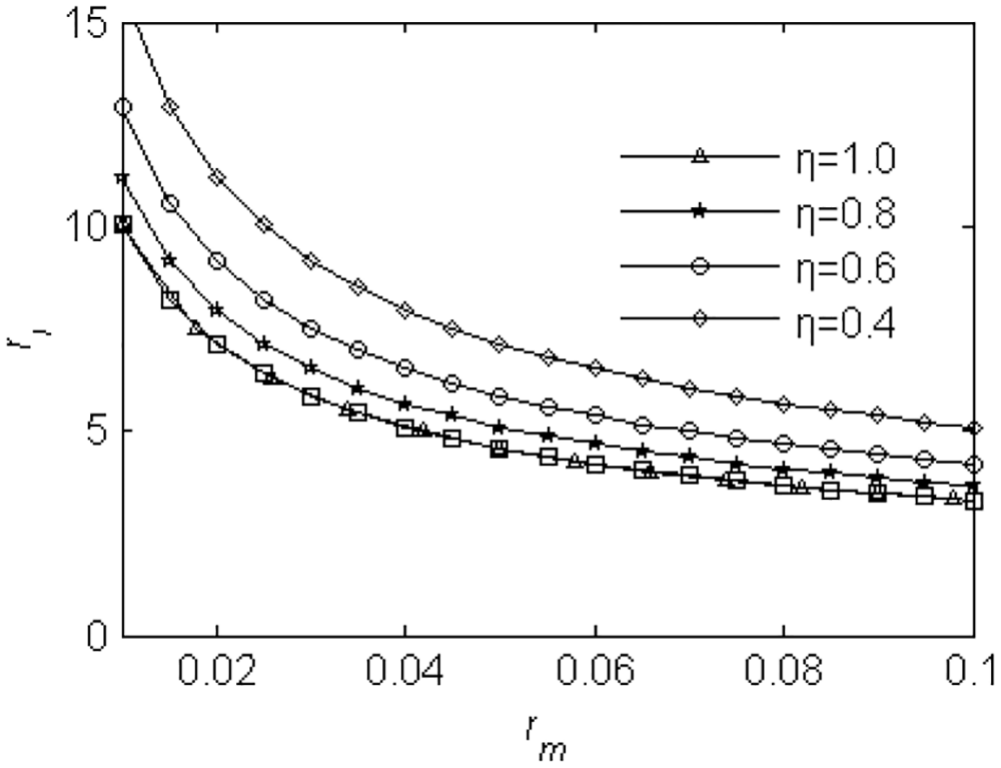
The value of parameter *r*_l_.

If $r_{l\max}^{2}$ satisfies Eqs (22) and (30), the synchronization of the two unbalance rotors can rotate stably. As shown in Fig 3, *r*_*lmax*_ = 7 for *η* = 1 and *r*_*m*_ = 0.02, the value of *r*_*l*_ can be confined range from zero to seven in the following discussions.

According to Ref. [22], when an induction motor rotate with the synchronous velocity *ω*_*m*_, its electromagnetic torque and stiffness coefficients of the angular velocity, *T*_*e0k*_ and *k*_*ek*_ (*k* = 1,2), can be simplified as

$$T_{ek}=n_{p}\frac{L_{mk}U_{S0}^{2}}{L_{sk}^{2}\omega_{s}R_{rk}}(\omega_{s}-n_{p}\omega_{m0})$$ (33)

$$k_{ek}=n_{p}^{2}\frac{L_{mk}^{2}U_{S0}^{2}}{L_{sk}^{2}\omega_{s}R_{rk}}$$ (34)

Where *L*_*mk*_ is the mutual inductance of the *k* ^*th*^ induction motor; *L*_*sk*_ is stator inductance of the *k* ^*th*^ induction motor;*n*_*p*_ is the number of pole pairs of the induction motor; *ω*_*s*_ is synchronous electric angular velocity; *R*_*rk*_ is the rotor resistance of the *k* ^*th*^ induction motor; *U*_*S0*_ is the amplitude of the stator voltage vector.

Substituting the above values of these parameters into the Eq (21), we can ascertain the value of the stable phase difference between the two rotors. To guarantee the synchronous operation of the two rotors, *T*_*C*_ in Eq (21) should be larger than parameter |*T*_*D*_|. When the two identical motors are employed to drive the two identical unbalanced rotors, we have

$$T_{D}=T_{R1}-T_{R2}=m_{1}r^{2}\omega_{m}^{2}(W_{s2}-W_{s1})/2$$ (35)

Here, we assume that *T*_*e01*_−f_*1*_ *ω*_*m*_−(*T*_*e02*_−f_*2*_ *ω*_*m*_)≈0 just for the convenient discussions. Actually, in engineering the difference between the electromagnetic torques of two identical motor is not equal to zero. Eq (22), therefore, can be simplified in the form

$${W^{\prime }}_{c}\geq \left|(W_{s2}-W_{s1})/2\right|$$ (36)

**Fig 4:**
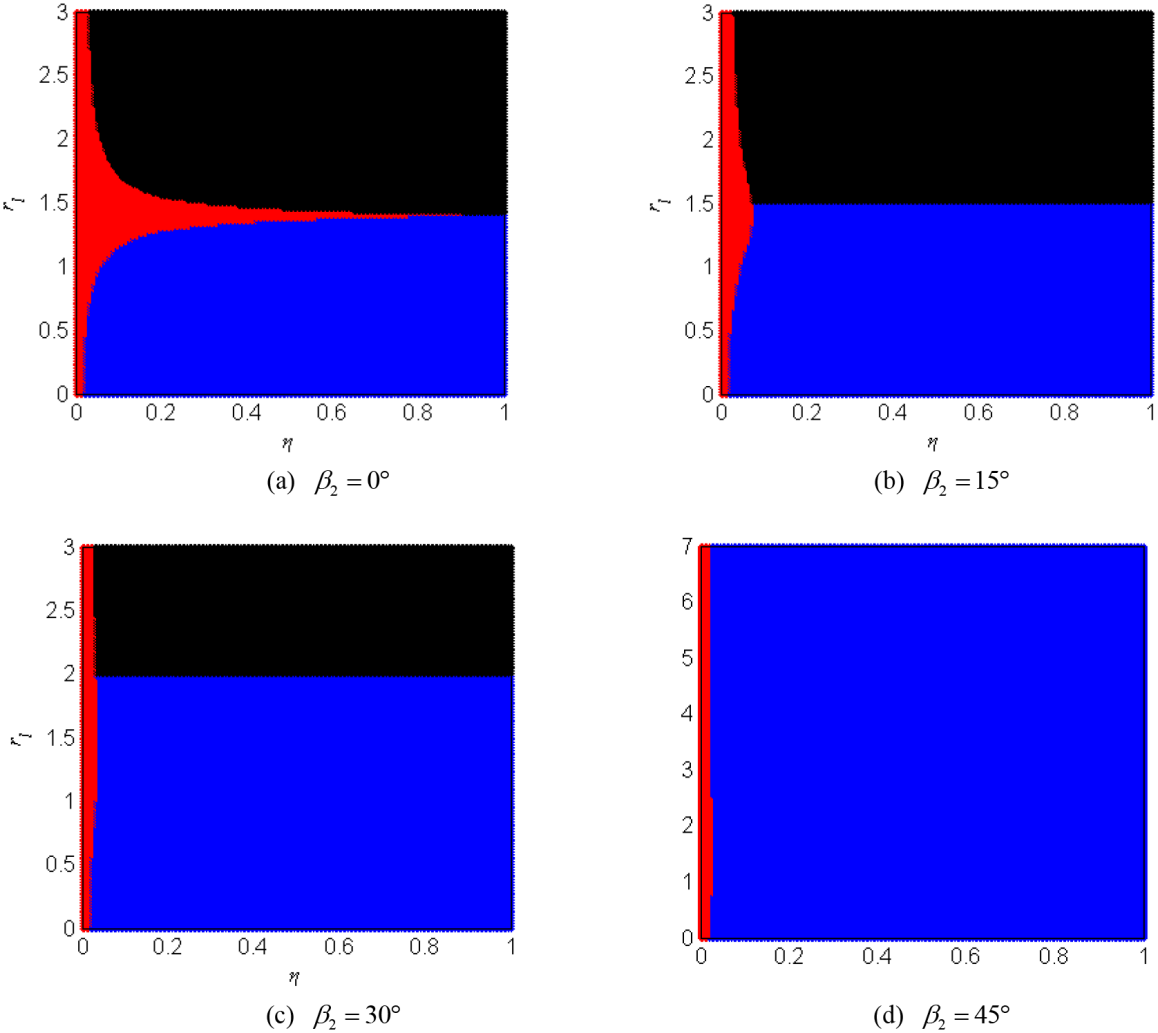
Synchronization region for the two unbalanced rotors.

According to Eq (36), we can sketch the synchronization regions for the vibration system. Fig 4 shows the region of implementing synchronous rotation between the two unbalanced rotors for the different value of the parameters. These figures are divided into a blue region, a black region, and a red region, respectively. If the value of the parameters of the vibration system locates in the blue region, the value of the stable phase difference between the two unbalanced belongs to the interval of [*π*/2,3*π/*2]. If the value of the parameters of the vibration system locates in the black region, the value of the stable phase difference between the two unbalanced belongs to the interval of [−*π*/2,*π/*2]. If the value of the parameters of the vibration system locates in the red region, not satisfying the synchronization condition, the two rotors cannot operate synchronously. Fig 4(a) describes the synchronization region in *ηr*_*l*_-plane for *β*_2_ = 0°. It can be seen that the system cannot implement synchronization when the value of *r*_*l*_ is equal to 1.414, in the case, there are ${W^{\prime }}_{c}=0$ and ${W^{\prime }}_{c}<\left|(W_{s2}-W_{s1})/2\right|$. By increasing the value of parameter *β*_2_, we find that the region of the red color and the black color are shrined. Especially, the black color region is disappeared when the value of parameter *β*_2_ approach to 45°. In this case, the value of *a*_*c1*_ and *a*_*c2*_ is less than zero, and so value of the phase difference only located in the interval of [*π*/2,3*π/*2]. As a result, it is obviously demonstrated that parameters *η* and *β*_2_ have influence on the synchronization regions of the vibration system.

**Fig 5:**
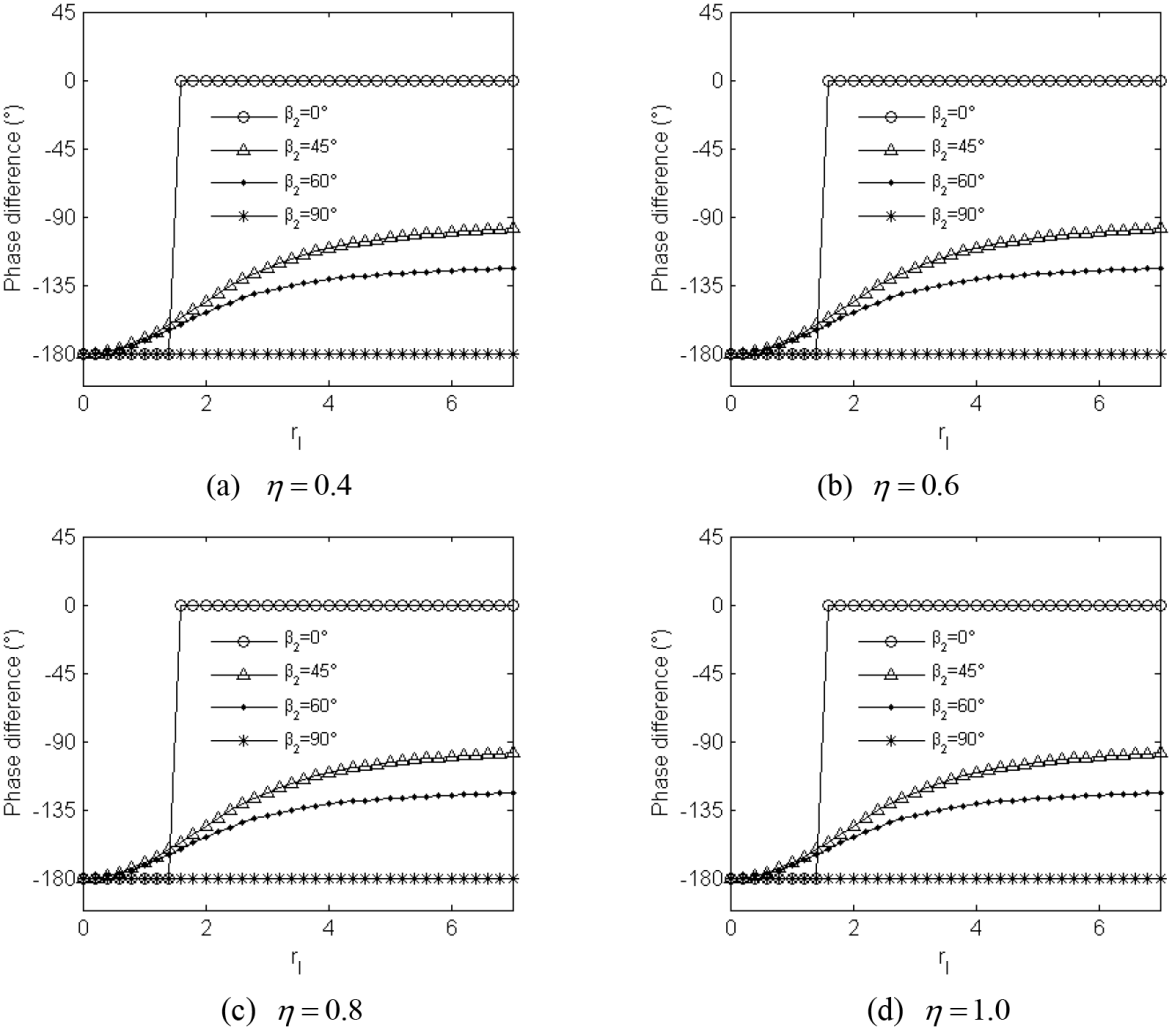
The approximate theoretical value of the stable phase difference for the identical coupling coefficients.

Eq (21) describes the approximate analytical solution for the stable phase difference. Base on the equation and Appendix B in S1 File, we can acquire the approximate value 2*α* considering the different value of parameter *η* with the identical coupling coefficients in *x*_*i*_, *y*_*i*_ and *ψ*_*i*_ direction between the two vibro-bodies (i.e. *μ*_*xij*_ = *μ*_*yij*_ = *μ*_*ψij*_, *i* = 1,2 *j* = 1,2), as shown in Fig 5. From Fig 5(a)–5(d), it can be seen that the value of parameter *η* has little influence on the value of the phase difference 2*α* when the parameters of the system satisfy the above-mention synchronization condition and synchronization stability criterion. But dimensionless parameters *r*_*l*_ and *β*_2_ directly determine the value of 2*α*: If *β*_2_ = 0° and *r*_*l*_<1.414, the phase difference 2*α* approximately stabilize at −180°(*a*_*c*_<0 in the case); if *β*_2_ = 0° and *r*_*l*_<1.414, the phase difference 2*α* approximately stabilize at 0° (*a*_*c*_>0 in the case). If *β*_2_ = 60°, the phase difference 2*α* gradually increase from −180° with increasing the value of *r*_*l*_ (*a*_*c*_>0 in the case). If *β*_2_ = 90°, the phase difference 2*α* always stabilize at −180° irrelevant to the value of *r*_*l*_ (*a*_*c*_>0 in the case). According to Appendix A in S1 File, parameter *r*_*l*_ is the function of parameters *l* and *l*_*o*_, and it can be concluded that the value of the phase difference 2*α* is determined by the installation position of the two induction motors (i.e. parameters *l* and *β*_2_).

**Fig 6:**
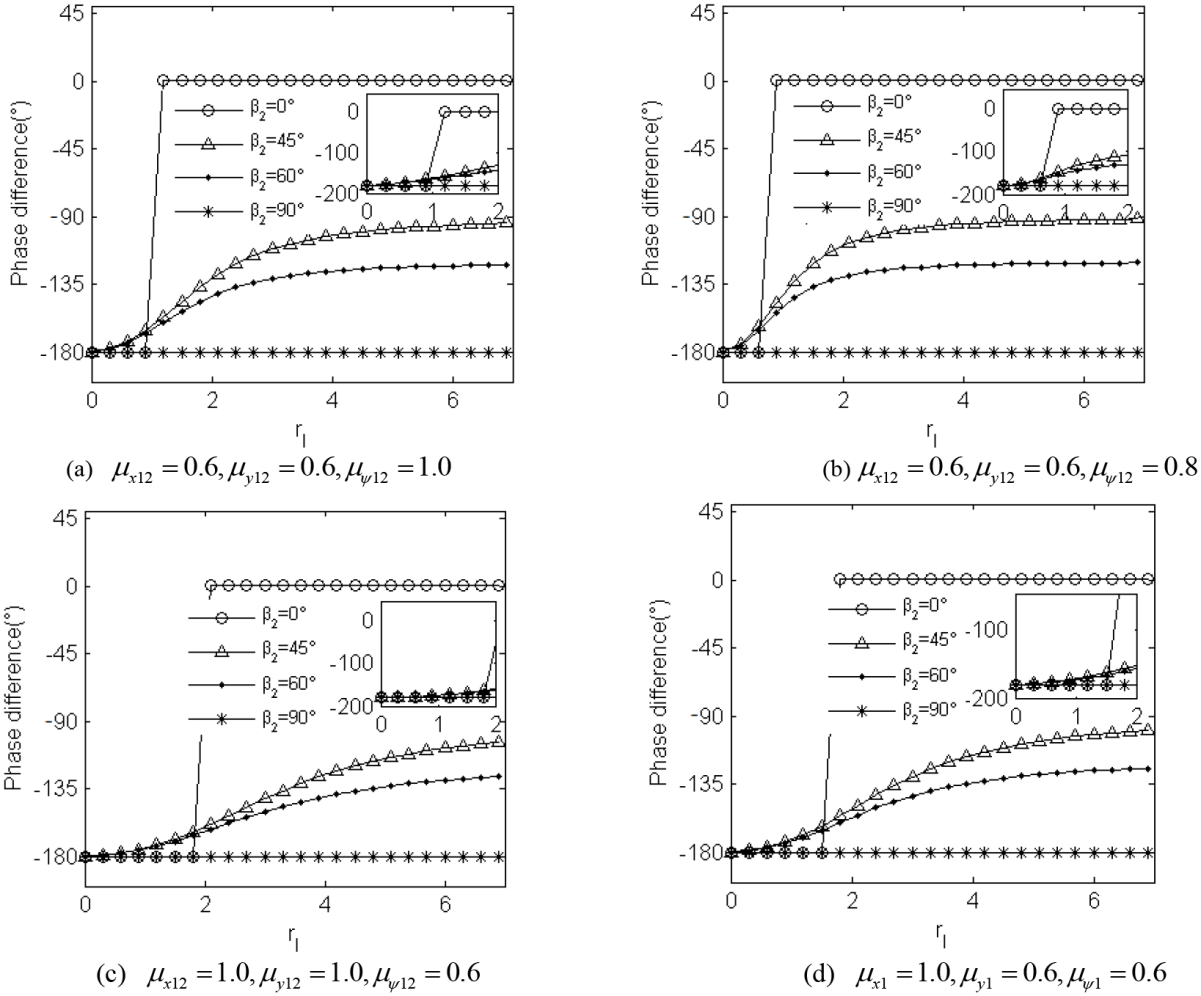
The approximate theoretical value of the stable phase difference for the non-identical coupling coefficients.

The analysis above imply that parameter *η* slightly affect the value of the stable phase difference, and here we numerically discuss the value of the phase difference considering the non-identical coupling coefficients with ignoring the variable of parameter *η*. Fig 6 describes the approximate analytical solution for the stable phase difference the non-identical coupling coefficients in the *x*_*i*_, *y*_*i*_ and *ψ*_*i*_-direction (*i* = 1,2.). Comparing with Fig 5, it can be found that the change of value of the non-identical coupling coefficients weakly effect on the value of the stable phase difference. And in the case of *β*_2_ = 90°, the value of the phase difference invariably stabilize at −180° irrelevant to the value of the coupling coefficients between the two vibro-bodies.

## Sample Validations

Further analyses have been performed by computer simulations to verify our above theoretical solutions, which can be carried out by applying the Runge-Kutaa routine with adaptive stepsize control to the dynamics equations of the proposed Eq (10).

Here, the parameters of the two motors are assumed to be the same (Three-phase squirrel-cage, i.e., Rated power 0.7 Kw, Rated voltage 220 V, Rated frequency 50Hz, Pole pairs 2, Stator resistance 0.56Ω, Rotor resistance 0.54 Ω, Stator inductance 0.1 H, Rotor inductance 0.12H, Mutual inductance 0.13 H, The damping coefficient of shafting 0.04 Nm/ (rad/s)).

The value of the parameters of the vibration system are: *m*_*1*_ = *m*_*2*_ = 5kg, *M*_*1*_ = *M*_*2*_ = 50kg, *r* = 0.04m, *l*<0.7m (for *r*_*l*_ ∈[0,7]), *k*_*x*1_ = *k*_*y*1_ = 2310kN/m, *k*_*ψ*1_ = 231kN m/rad (*n*_*i*1_ = 1.0), *k*_*x*2_ = *k*_*y*2_ = 250kN/m, *k*_*ψ*2_ = 25kN m/rad (*n*_*i*2_ = 3), *J*_*1*_ = *J*_*2*_ = 10kg m^2^, *f*_*x*1_ = *f*_*y*1_ = *f*_*x*2_ = *f*_*y*2_ = 4.85kN s/m, *f*_*ψ*1_ = *f*_*ψ*2_ = 0.54kN s/m.

### Simulation results for *η =* 1.0, r_*l*_ = 6 and *β*_2_ = 0°

**Fig 7:**
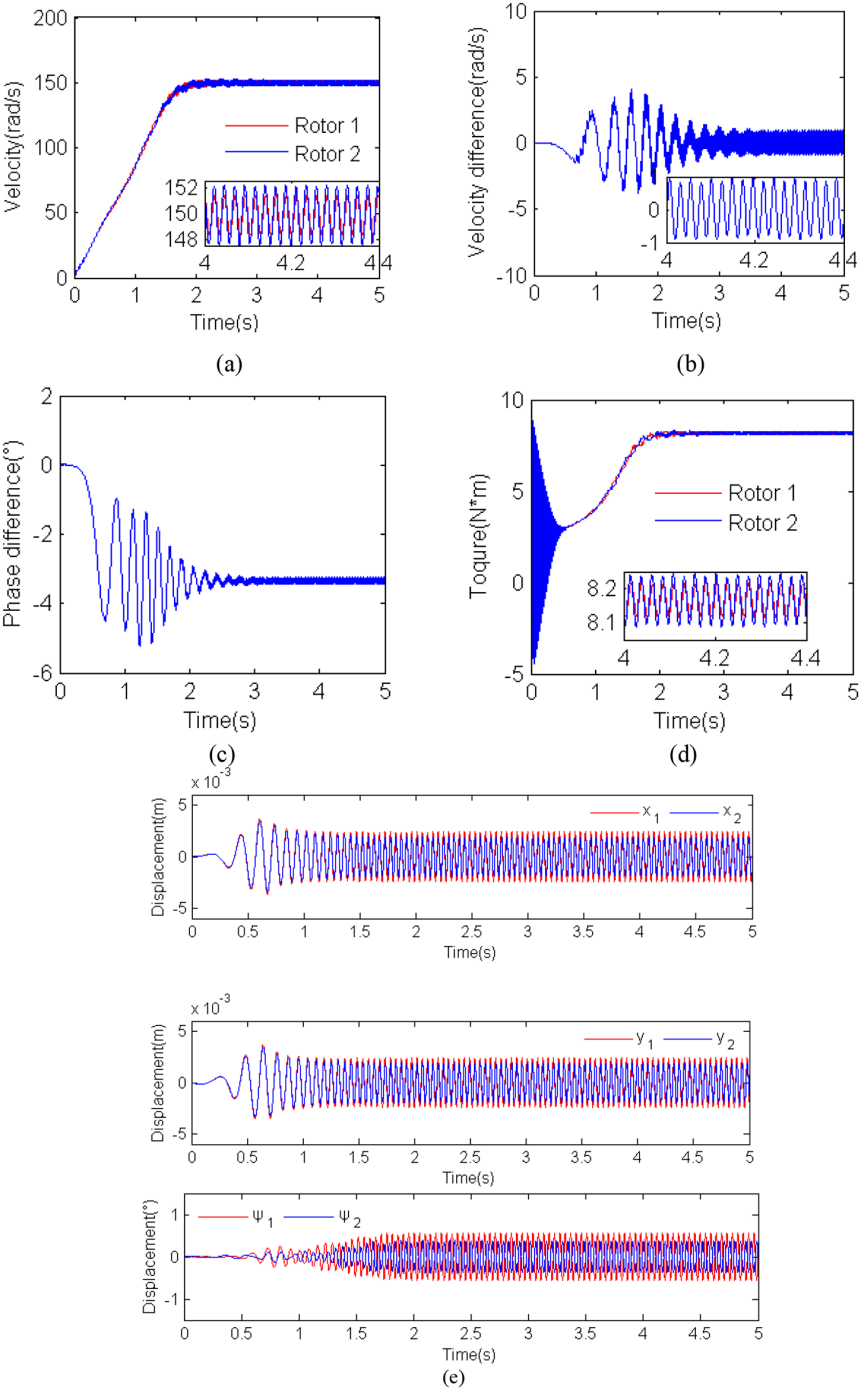
Results for the computer simulation for *η* = 1.0, *r_l_* = 6 and *β*2 = 0° . **(a) rotational velocities of the two induction motors; (b) velocity difference of the two motors; (c) phase difference between the two unbalanced rotors; (d) electromagnetic torques of the two motors; (e) displacement responses of the vibro-body in the DOFs.**

Simulation results for *η =* 1.0, r_***l***_ = 6 and *β*_2_ = 0° (i.e., *m*_*1*_ = *m*_*2*_ = 5Kg, *r*_*m*_ = 0.02m, *β*_1_ = 180° and *l* = 0.8m) as shown in Fig 7. When the two motors are supplied with the electric source at the same time, the angular accelerations of the two motor are approximately equal to each other because the inertia moments of two rotors are identical, as illustrate in Fig 7(a). After a few seconds, the rotational velocity of the two motors approach a steady velocity. In addition, the high frequency vibration of the vibro-bodies is excited and the load torques of the motors on which the displacement of the vibro-body is larger, therefore, that makes the two unbalanced rotors synchronous. To ensure the synchronous rotation of the rotors, the motors should provide the equal electromagnetic torques to overcome the load torques. In the case, the electromagnetic torques of the motors should be identical.

From Fig 7(a)–7(d) it follows that the steady synchronization of the system is implemented at about 2 s. In the synchronization state, the two unbalanced rotors operate with approximate velocity 150 rad/s, which is called the synchronous velocity; the velocity difference ranges from -1 to 1 rad/s; the phase difference 2*α* stabilized in the vicinity of −3.5°, which is according with the approximate values of the theory analysis, as shown in Fig 5(d); the electromagnetic torques of the two motors stabilized at 8.15 Nm and are identical each other. It should be noted that, in this case, the value of the parameters of the vibration system located in the blue region, as shown in Fig 4(a). The displacement responses of the vibro-bodies in the DOFs are sketched in Fig 7(e). It can be seen that the displacement in *x*_1_, *x*_2_, *y*_1_ and *y*_2_-direction is larger than 0.0025 m as the phase difference −3.5° between the rotors leads to the addition of the exciting forces through the coupling springs supporting vibro-body-1. And the displacement amplitude of the vibro-body-1 is slightly larger than the vibro-body 2.

### Simulation results for *η =* 1.0, r_*l*_ = 0.95 and *β*_2_ = 90°

**Fig 8:**
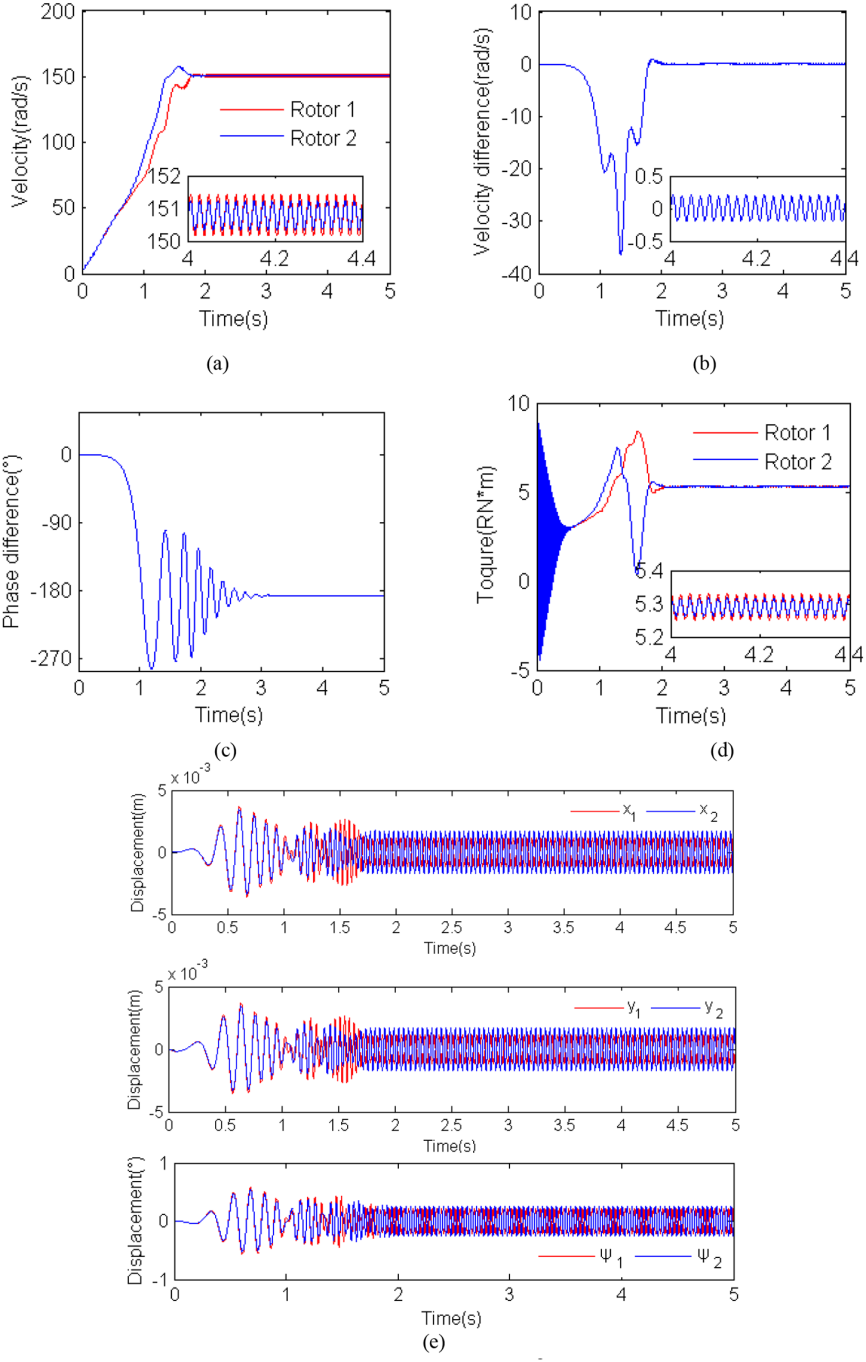
Results for the computer simulation for *η =* 1.0, *r_l_* = 0.95 and *β*_2_ = 90° . **(a) rotational velocities of the two induction motors; (b) velocity difference of the two motors; (c) phase difference between the two unbalanced rotors; (d) electromagnetic torques of the two motors; (e) displacement responses of the vibro-body in the DOFs.**

Fig 8 shows simulation results for *η =* 1.0, r_*l*_ = 0.95 and *β*_2_ = 90° (i.e., *m*_*1*_ = *m*_*2*_ = 5Kg, *r* = 0.02m, *β*_1_ = 90° and *l* = 0.2m). During the starting few seconds, the angular accelerations of the two motor are also approximately equal to each other. After about 2 s, the two rotors rotate with synchronization velocity 150 rad/s; the velocity difference ranges from -0.5 to 0.5 rad/s; the phase difference 2*α* stabilized in the vicinity of 180°, which is coincident with the approximate values of the theory analysis, as shown in Fig 5(a); the electromagnetic torques of the two motors stabilized at 5.3Nm and are also identical each other. Fig 8(e) shows displacement responses of the vibro-bodies in the DOFs. It is noted that the displacement in *x*_1_, *x*_2_, *y*_1_ and *y*_2_-direction is less than 0.0025 m as the phase difference 180° leads to the offset of the part of the exciting forces through springs supporting vibro-body-1. The displacement amplitude of the vibro-body-1 is slightly smaller than the vibro-body-2.

### Simulation results for *η =* 0.2, r_*l*_ = 1.3 and *β*_2_ = 0°

**Fig 9:**
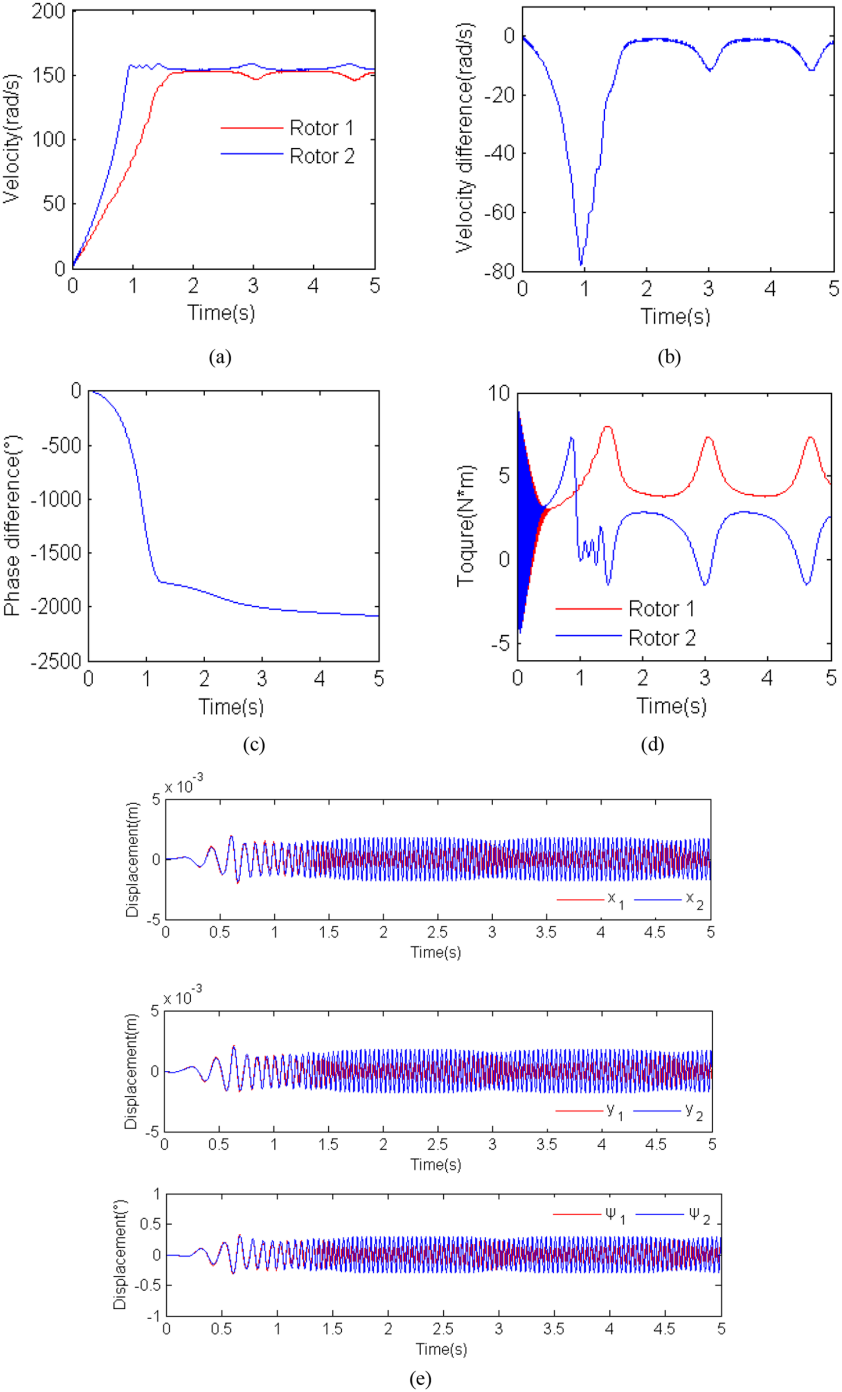
Results for the computer simulation for η = 0.2, *r_l_* = 1.3 and *β*_2_ = 0°. (a) rotational velocities of the two induction motors; (b) velocity difference of the two motors; (c) phase difference between the two unbalanced rotors; (d) electromagnetic torques of the two motors; (e) displacement responses of the vibro-body in the DOFs.

To further verify the theory analysis results for absent synchronization of the rotor considering parameter *η*, it is necessary for two non-identical to perform computer simulations, and the results are shown in Fig 9. Here, *η =* 0.2, *β*_2_ = 0° and r_*l*_ = 1.3 (i.e., *m*_*1*_ = 5Kg, *m*_*2*_ = 1kg, *β*_1_ = 180° and *l* = 0.3). During the starting few seconds, the angular velocity of rotor 1 is far less than rotor 2. The reason is that the inertia moment of rotor 1 is greater than rotor 2. After a few seconds the rotational velocity of two rotors approach the high rotation and absent synchronization rotation, in addition, the maximum value of the velocity difference between the two rotors exceed 10 rad/s, as shown in Fig 9(a)–9(b). Meanwhile, the phase difference cannot be stable because of the non-identical velocity of the induction motors, as shown in Fig 9(c). In this case, the value of the parameters of the vibration system is located in the red region as shown in Fig 4(a), and the electromagnetic torque of the motors is unstable and unequal with the different load torque on the shaft of the motors as shown in Fig 9(d). From Fig 9(e), it follows the displacement responses of the vibro-bodies. It can be seen that the displacement responses of the vibro-bodies is unstable because of the unstable phase difference and the fluctuation of the motors’ velocity. The system within such parameters cannot be suitably applied in the engineering.

## Conclusions

The vibration system we proposed in this article could be used to invent new vibration screens for the solid-liquid separation in the offshore drilling engineering when their parameters satisfy the synchronization condition and the synchronization stability criterion. Due to the development in the early stage and clearly understand the operation mechanism, we consider the case that the stronger stiffness springs between the two vibro-bodies is coupled with the weaker stiffness springs connected with the fixed foundation. In the future researches, considering the other coupling styles of the system and the unbalanced rotors drove with Multi-motors will be interesting for the exploration of the vibration screens. With the theoretical investigation and the numerical simulations, the following conclusions are obtained:

The average method of modified small parameters is used to simplify mathematically the deducing process. Base this method, the non-dimensionless coupling equations of the vibration system are acquired, and then, the problem of synchronization is converted into that of existence and the stability of zero solutions for the non-dimensional differential equations of the angular velocity disturbance parameters. The synchronization condition for the two rotors is that the absolute value of the residual torque between the two motors is equal to or less than the maximum of their coupling torques. When the value of the parameters of the system is located in the blue region of synchronization, the two unbalanced rotor can implement the synchronous rotation. With the Routh-Hurwitz criterion, the region of the stable phase difference is confirmed by parameter *θ*_*c*_, obviously, it is demonstrated that parameters *η*, *β*_1_, *β*_2_ and r_*l*_ have an influence on the synchronization regions of the vibration system. At last, computer simulations is preformed to verify the correctness of the approximate solution of the theoretical computation for the stable difference between the two unbalanced rotors, and the results of theoretical computation is in accordance with that of computer simulations.

## Supporting Information

S1 File Supplementary Appendices. S1 File include appendix A and B. Appendix A describes the solutions for the steady responses of the vibration system, and appendix B describes the coefficients of Eq (15).
(DOCX)
