# Supplementary material for: Synchronization of Two Homodromy Rotors Installed on a Double Vibro-Body in a Coupling Vibration System
Source: PLoS One. 2015 May 19;10(5):e0126069. doi: 10.1371/journal.pone.0126069 (PMC4437654; doi:10.1371/journal.pone.0126069)
Supplement: S1 File — include appendix A and B. Appendix A describes the solutions for the steady responses of the vibration system, and appendix B describes the coefficients of Eq (15). (DOCX) [file pone.0126069.s001.docx]

# Supplementary Appendices

**Appendix A: The solutions for the steady responses of the vibration system**

The steady response solutions of the DOFs can be expressed as

Where

$$\begin{matrix} i=x,y,\psi, \\ j=x,y, \\ a_{i}=(1-n_{i1}^{2})(1-n_{i2}^{2})-n_{i2}(\sigma_{12}n_{i2}+4\xi_{i1}\xi_{i2}n_{i1}), \\ b_{i}=2\xi_{i1}n_{i1}(1-\sigma_{12}n_{i2}^{2}-n_{i2}^{2})+2\xi_{i1}n_{i2}(1-n_{i1}^{2}), \\ g_{i1}=-n_{i1}^{2}n_{i2}^{2}+\sigma_{12}n_{i2}^{2}+n_{i1}^{2},h_{i1}=2n_{i1}n_{i2}(\sigma_{12}\xi_{i1}n_{i2}+\xi_{i2}n_{i1}), \\ o_{i1}=n_{i2}^{2},p_{i1}=2\xi_{i1}n_{i2}^{2}n_{i1}, \\ g_{i2}=-n_{i1}^{2}n_{i2}^{2}+n_{i2}^{2},h_{i2}=2\xi_{ni1}n_{i1}n_{i2}^{2}, \\ o_{i2}=n_{i2}^{2},p_{i2}=2\xi_{i1}n_{i1}^{2}n_{i2}, \\ \omega_{nj1}=\sqrt{k_{j1}/M_{1}},\xi_{j1}=f_{j1}/2\sqrt{M_{1}k_{j1}},n_{i1}=\omega_{m}/\omega_{ni1}, \\ \omega_{nj2}=\sqrt{k_{j2}/M_{1}},\xi_{j2}=f_{j1}/2\sqrt{M_{1}k_{j2}},n_{i2}=\omega_{m}/\omega_{ni2}, \\ \omega_{n\psi1}=\sqrt{k_{\psi1}/M_{1}l_{o}^{2}},\xi_{n\psi1}=f_{\psi1}/2\sqrt{M_{1}l_{o}^{2}k_{\psi1}}, \\ \omega_{n\psi2}=\sqrt{k_{\psi2}/M_{2}l_{o}^{2}},\xi_{n\psi2}=f_{\psi2}/2\sqrt{M_{2}l_{o}^{2}k_{\psi2}}, \\ m_{2}/m_{1}=\eta,m_{1}/M=r_{m},m_{2}/M=\eta r_{m}, \\ r_{l}=l_{o}/l\text{，}\beta_{1}=\pi-\beta_{2}, \\ \mu_{i11}=\sqrt{\frac{g_{i1}^{2}+h_{i1}^{2}}{a_{i}^{2}+b_{i}^{2}}},r_{i11}=tg^{-1}(\frac{b_{i}g_{i1}-a_{i}h_{i1}}{a_{i}g_{i1}+b_{i}h_{i1}}), \\ \mu_{i12}=\sqrt{\frac{o_{i1}^{2}+p_{i1}^{2}}{a_{i}^{2}+b_{i}^{2}}},r_{i12}=tg^{-1}(\frac{b_{i}o_{i1}-a_{i}p_{i1}}{a_{i}o_{i1}+b_{i}p_{i1}}), \\ \mu_{i21}=\sqrt{\frac{g_{i2}^{2}+h_{i2}^{2}}{a_{i}^{2}+b_{i}^{2}}},r_{i21}=tg^{-1}(\frac{b_{i}g_{i2}-a_{i}h_{i2}}{a_{i}g_{i2}+b_{i}h_{i2}}), \\ \mu_{i22}=\sqrt{\frac{o_{i2}^{2}+p_{i2}^{2}}{a_{i}^{2}+b_{i}^{2}}},r_{i22}=tg^{-1}(\frac{b_{i}o_{i2}-a_{i}p_{i2}}{a_{i}o_{i2}+b_{i}p_{i2}}). \end{matrix}$$

**Appendix B:** **The coefficients of Eq. (15)**

$$\begin{matrix} W_{c1}=r_{m}(\mu_{x11}\cos\gamma_{x11}+\mu_{y11}\cos\gamma_{y11}+r_{l}^{2}\mu_{\psi11}\cos r_{\psi11}) \\ W_{s1}=r_{m}(\mu_{x11}\sin\gamma_{x11}+\mu_{y11}\sin\gamma_{y11}+r_{l}^{2}\mu_{\psi11}\sin r_{\psi11}) \\ W_{c2}=\eta^{2}r_{m}(\mu_{x21}\cos r_{x21}+\mu_{y21}\cos r_{y21}+r_{l}^{2}\mu_{\psi21}\cos r_{\psi21}) \\ W_{s2}=\eta^{2}r_{m}(\mu_{x21}\sin r_{x21}+\mu_{y21}\sin r_{y21}+r_{l}^{2}\mu_{\psi21}\sin r_{\psi21}) \end{matrix}$$

$$\begin{matrix} W'_{c1}=\eta r_{m}\sqrt{a_{c1}^{2}+b_{c1}^{2}} \\ a_{c1}=-\mu_{x12}\cos\gamma_{x12}-\mu_{y12}\cos\gamma_{y12}+r_{l}^{2}\mu_{\psi12}\cos\gamma_{\psi12}\cos(2\beta_{2}) \\ b_{c1}=-r_{l}^{2}\mu_{\psi12}\cos\gamma_{\psi12}\sin(2\beta_{2}) \\ \theta_{c1}=\left\{\begin{matrix} \arctan(-\frac{b_{c1}}{a_{c}}),a_{c1}\geq0, \\ \pi+\arctan(-\frac{b_{c1}}{a_{c1}}),a_{c1}\leq0. \end{matrix}\right. \end{matrix}$$

$$\begin{matrix} W'_{c2}=\eta r_{m}\sqrt{a_{c2}^{2}+b_{c2}^{2}} \\ a_{c2}=-\mu_{x22}\cos r_{x22}-\mu_{y22}\cos r_{y22}+r_{l}^{2}\mu_{\psi22}\cos r_{\psi22}\cos(2\beta_{2}) \\ b_{c2}=-r_{l}^{2}\mu_{\psi22}\cos r_{\psi22}\sin(2\beta_{2}) \\ \theta_{c2}=\left\{\begin{matrix} \arctan(-\frac{b_{c2}}{a_{c2}}),a_{c2}\geq0, \\ \pi+\arctan(-\frac{b_{c2}}{a_{c2}}),a_{c2}<0. \end{matrix}\right. \end{matrix}$$

$$\begin{matrix} W'_{s1}=\eta r_{m}\sqrt{a_{s1}^{2}+b_{s1}^{2}} \\ a_{s1}=\mu_{x12}\sin\gamma_{x12}+\mu_{y12}\sin\gamma_{y12}+r_{l}^{2}\mu_{\psi12}\sin\gamma_{\psi12}\cos(2\beta_{2}) \\ b_{s1}=-r_{l}^{2}\mu_{\psi12}\sin\gamma_{\psi12}\sin(2\beta_{2}) \\ \theta_{s1}=\left\{\begin{matrix} \arctan(\frac{b_{s1}}{a_{s1}}),a_{s1}\geq0, \\ \pi+\arctan(\frac{b_{s}}{a_{c}}),a_{s1}\leq0. \end{matrix}\right. \end{matrix}$$

$$\begin{matrix} W'_{s2}=\eta r_{m}\sqrt{a_{s2}^{2}+b_{s2}^{2}}\sin(2\alpha+\theta_{s2}) \\ a_{s2}=\mu_{x22}\sin r_{x22}+\mu_{y22}\sin r_{y22}+r_{l}^{2}\mu_{\psi22}\sin r_{\psi22}\cos(2\beta_{2}) \\ b_{s2}=-r_{l}^{2}\mu_{\psi22}\sin r_{\psi22}\sin(2\beta_{2}) \\ \theta_{s2}=\left\{\begin{matrix} \arctan(\frac{b_{s2}}{a_{s2}}),a_{s2}\geq0, \\ \pi+\arctan(\frac{b_{c2}}{a_{c2}}),a_{s2}<0. \end{matrix}\right. \end{matrix}$$
